# Supplementary material for: Designing a Library of Lived Experience for Mental Health: integrated realist synthesis and experience-based co-design study in UK mental health services
Source: BMJ Open. 2024 Jan 31;14(1):e081188. doi: 10.1136/bmjopen-2023-081188 (PMC10831458; doi:10.1136/bmjopen-2023-081188)
Supplement: Supplementary data [file bmjopen-2023-081188supp001.pdf]

TITLE: Designing a Library of Lived Experience for Mental Health: integrated realist synthesis and experience-based co-design study

Supplemental file 1 – COREQ checklist

No. Item	Guide questions/description	Reported on Page #
**Domain 1: Research team and reflexivity**		
Personal Characteristics		
1. Inter viewer/facilitator	Which author/s conducted the interview or focus group?	Interviews were conducted by the lead author (PM) (page 5 line 19)
2. Credentials	What were the researcher's credentials? E.g. PhD, MD	PhD (corresponding author information)
3. Occupation	What was their occupation at the time of the study?	Research Associate (page 5 line 19)
4. Gender	Was the researcher male or female?	Male (page 5 line 22)
5. Experience and training	What experience or training did the researcher have?	Doctoral training in qualitative methods (page 5 line 20)
Relationship with participants		
6. Relationship established	Was a relationship established prior to study commencement?	Interviewees were members of a study advisory group and co-authors and as such, there was an established relationship with the researcher conducting the interview (page 5 line 21-22).
7. Participant knowledge of the interviewer	What did the participants know about the researcher? e.g. personal goals, reasons for doing the research	Interviewees were fully informed of the nature of the study prior to participation, including the researcher's aims for the interview and research study more broadly (page 5 line 21-22)
8. Interviewer characteristics	What characteristics were reported about the inter viewer/facilitator? e.g. Bias, assumptions, reasons and interests in the research topic	The interviewer had no prior experience of living libraries in any capacity and bias was mitigated through feedback from an advisory group with diverse experiences of living libraries (page 5 lines20-25)
Domain 2: study design		
Theoretical framework		
9. Methodological orientation and Theory	What methodological orientation was stated to underpin the study? e.g. grounded theory, discourse analysis, ethnography, phenomenology, content analysis	This study was underpinned by a realist methodological orientation (page 4, line 35)
Participant selection		

TITLE: Designing a Library of Lived Experience for Mental Health: integrated realist synthesis and experience-based co-design study

10. Sampling	How were participants selected? e.g. purposive, convenience, consecutive, snowball	Interviewees and workshop participants were purposively sampled based on their experience of living libraries (interviews, page 5, line 16) and mental health experiences (workshops, page 6, line 14-15)
11. Method of approach	How were participants approached? e.g. face-to-face, telephone, mail, email	Digital study advertisements (page 6, line 12)
12. Sample size	How many participants were in the study?	7 interviews (page 5, line 15) and 31 workshop participants (page 7, line 14)
13. Non-participation	How many people refused to participate or dropped out? Reasons?	Workshop retention is reported in Table 1 (page 8, line 22) – reasons for non-attendance were not recorded
Setting		
14. Setting of data collection	Where was the data collected? e.g. home, clinic, workplace	Data was collected online via video call. Page 5, line 19 (interviews), page 6, line 21 (workshops)
15. Presence of non-participants	Was anyone else present besides the participants and researchers?	No other people were present for interviews. Workshops included several co-facilitators described on page 6, lines 17-18 workshops).
16. Description of sample	What are the important characteristics of the sample? e.g. demographic data, date	Workshop demographics are provided in supplemental file 6. Interviewee demographic characteristics were not recorded.
Data collection		
17. Interview guide	Were questions, prompts, guides provided by the authors? Was it pilot tested?	Interview topic guide is provided in supplemental file 2.
18. Repeat interviews	Were repeat inter views carried out? If yes, how many?	No
19. Audio/visual recording	Did the research use audio or visual recording to collect the data?	Interviews were audio recorded (page 5, line 19). Workshops were not audio recorded in order to promote participants' feelings of safety in engaging with group discussions about mental health.
20. Field notes	Were field notes made during and/or after the interview or focus group?	Researcher field notes were generated during co-design workshops (page 6 lines 29). Not applicable to interviews

TITLE: Designing a Library of Lived Experience for Mental Health: integrated realist synthesis and experience-based co-design study

21. Duration	What was the duration of the interviews or focus group?	Specific online call duration was not recorded.
22. Data saturation	Was data saturation discussed?	No – data saturation is typically not discussed in this form of analysis
23. Transcripts returned	Were transcripts returned to participants for comment and/or correction?	No – however, analytic outputs were (page 6, lines 33-36)
Domain 3: analysis and findings		
Data analysis		
24. Number of data coders	How many data coders coded the data?	3 – PM, FL, and RJ (Page 6, lines 5-6)
25. Description of the coding tree	Did authors provide a description of the coding tree?	No
26. Derivation of themes	Were themes identified in advance or derived from the data?	From the data (page 5, lines 3-5)
27. Software	What software, if applicable, was used to manage the data?	NVivo 12 (page 6, line 31)
28. Participant checking	Did participants provide feedback on the findings?	Yes – written and verbal feedback from an advisory group (page 56, lines 33-36)
Reporting		
29. Quotations presented	Were participant quotations presented to illustrate the themes/findings? Was each quotation identified? e.g. participant number	No – however, deidentified workshop data were used to illustrate findings
30. Data and findings consistent	Was there consistency between the data presented and the findings?	In order to demonstrate this consistency, findings are presented with reference to extracts of underlying data from relevant literature and workshops (Table 2 – page 11, line 11)
31. Clarity of major themes	Were major themes clearly presented in the findings?	A detailed description of each major theme, or context-mechanism-outcome configuration (CMO), is presented (page 8 line 8 – page11 line 9)
32. Clarity of minor themes	Is there a description of diverse cases or discussion of minor themes?	Not applicable to all themes, however, diverse experiences of safety and potential harms are described in CMO 1 (page 8 line 10 – page 9 line 10)

TITLE: Designing a Library of Lived Experience for Mental Health: integrated realist synthesis and experience-based co-design study

Adapted from: Tong, A., Sainsbury, P., & Craig, J. (2007). Consolidated criteria for reporting qualitative research (COREQ): a 32-item checklist for interviews and focus groups. *International journal for quality in health care*, *19*(6), 349-357.
