# Supplementary material for: Designing a Library of Lived Experience for Mental Health: integrated realist synthesis and experience-based co-design study in UK mental health services
Source: BMJ Open. 2024 Jan 31;14(1):e081188. doi: 10.1136/bmjopen-2023-081188 (PMC10831458; doi:10.1136/bmjopen-2023-081188)
Supplement: Supplementary data [file bmjopen-2023-081188supp002.pdf]

TITLE: Designing a Library of Lived Experience for Mental Health: integrated realist synthesis and experience-based co-design study

Supplemental file 2 – Interview topic guide

The topic guide should be amended to reflect the interviewees' experiences of living libraries as books/readers/organisers/researchers. In section 1, focus on developing an understanding of participant experiences by using open ended, exploratory questioning. In section 2, if applicable, use the developing programme theory to further explore participants' experiences with reference to the specific hypotheses being tested at that stage of theory development.

Section 1: Participant experiences of living libraries

Suggested topics and example questions (select as appropriate). These topics remain flexible as per the semi-structured interview method.

Topic 1: Type of event and context

- Did you attend as a book/reader/organiser?
- What was the purpose of the living library event you attended/organised?
- How did you find out/how were you recruited/how did you recruit for the event?
- What was the format of the event? In-person/online? Time restricted conversations? One off or recurring events?
- Were you given instructions prior to the event/did you provide training for attendees?

Topic 2: Sharing experiences

TITLE: Designing a Library of Lived Experience for Mental Health: integrated realist synthesis and experience-based co-design study

- Did you have any views/expectations about sharing/hearing a story before the event?
- How did you find out about the books that were available to speak with/provide options for readers?
- How did you decide who to speak to/when?
- What was the format of the discussions?

Topic 3: Impacts

- How did you experience sharing your story/hearing someone else's story?
- What was it like speaking with someone about [the topic] in particular?
- Were there unexpected effects of taking part?
- What effect (if any) did the living library event on your views of the topics discussed, and why?

Section 2: Theory testing

Following the guidance for realist interviews provided by Manzano (2016), prior to each interview, consider how the current programme theory can be further understood with reference to context, mechanism and outcome. Adapt the interview questions depending on the stage of theory development: in early interviews make greater use of general and exploratory questions to inform tentative theories; later, theory refinement focused interviews should increase in specificity and may draw on specific examples/cases to illustrate CMOs in discussions with participants; theory consolidation focuses on refining existing hypotheses, for example, how CMOs may vary depending on different stakeholder perspectives and how CMOs may link together across the programme theory.

TITLE: Designing a Library of Lived Experience for Mental Health: integrated realist synthesis and experience-based co-design study

Note: examples of how CMOs may be developed with a focus on theory development (adapted from Manzano, 2016):

Exploring context:

What characteristics of the living library location contribute to people feeling able to share their stories?

Looking for mechanisms:

How do you think the living library event influenced the attitudes of those who took part?

Looking for intended and unintended outcomes:

In your opinion, how should participants be supported when asked to share their experiences as part of a living library?

References

Manzano, A. (2016). The craft of interviewing in realist evaluation. *Evaluation*, *22*(3), 342-360.
