# Supplementary material for: Designing a Library of Lived Experience for Mental Health: integrated realist synthesis and experience-based co-design study in UK mental health services
Source: BMJ Open. 2024 Jan 31;14(1):e081188. doi: 10.1136/bmjopen-2023-081188 (PMC10831458; doi:10.1136/bmjopen-2023-081188)
Supplement: Supplementary data [file bmjopen-2023-081188supp003.pdf]

TITLE: Designing a Library of Lived Experience for Mental Health: integrated realist synthesis and experience-based co-design study

Supplemental file 3 - Initial programme theories

1. Readers – hearing stories about mental health

Intended impacts

1.1 Reduced isolation - If readers hear stories that resonate with their own experiences of mental health difficulties, then they will feel that their experiences are shared, thus normalising their experience and reducing their sense of social isolation. This shared experience may make them feel they are more likely to be understood by the book, and by others with similar experiences, and therefore more able to talk about their mental health difficulties with others, promoting social engagement and help-seeking.

1.2 Tailored learning - If readers take part in a library that facilitates synchronous interaction, then they will be able to ask the questions about mental health that they want answered, increasing the relevance of the information shared by the book to their personal experience and individual learning needs. This will mean readers are better informed and have the information they need to manage their own mental health difficulties.

1.3 Challenging preconceptions - If readers interact with a living book who through their participation in the library challenges the reader's negative preconceptions or stigma regarding mental health, for example that it is not possible to live well with a mental health problem, then contact with the book will disconfirm these preconceptions, increasing hope for their own recovery.

1.4 Connecting to the individual - If the reader engages with a book in a synchronous, face-to-face interaction, then this intimate interpersonal context facilitated will promote empathy with the book as their storytelling will be perceived as more authentic and emotive, facilitating other intended impacts (such as learning, social connection, and hope for recovery).

1.5 Connecting to the story - If the reader interacts with a book whose story involves a compelling narrative about their mental health difficulties, then the reader will be more engaged by their story, making the story more likely to be internalised and remembered, facilitating other impacts such as lasting change in perspective. This impact will be greater in a context where shared life experiences create personal resonance with the story.

Unintended impacts

TITLE: Designing a Library of Lived Experience for Mental Health: integrated realist synthesis and experience-based co-design study

1.6 Exposure to distressing stories - If the reader interacts with a book who recalls distressing aspects of their experiences and/or who becomes distressed when doing so, then the reader may be at increased risk of themselves becoming distressed.

1.7 Interpersonal tension - If the reader engages with a book who they perceive holds views that they find objectionable, then there is an increased risk of interpersonal tension and possible subsequent distress is increased The reader is also unlikely to identify with the book, and more likely to actively reject any strategies or ideas that the book offers, so will not learn anything of value and feel they have wasted their time. This could make them less likely to engage with other forms of peer-based support in future.

1.8 Negative social comparison - If the reader engages in negative social comparison, for example perceives that they are unlikely to achieve the level of recovery experienced by the living books at the library, then they may feel dejected and less hopeful about their own recovery, and therefore less likely to engage in recovery focussed activities. This is particularly likely to happen if the books are selected for "success" (or "recovery") stories only.

2. Living 'books' – telling stories about mental health

Intended impacts

2.1 Using experiences to help others - If living books tell their stories of mental health difficulties to readers who they feel have been derived benefit from hearing their experiences, those telling their stories will derive satisfaction from their participation contributing to a sense of personal meaning.

2.2 Developing personal understanding - If living books tell their stories of mental health difficulties, then the process of recalling and communicating their story in a safe environment may facilitate the development of new understandings of their previous experiences, contributing to greater awareness of their own mental health.

2.3 Social inclusion - Where living books are encouraged to use their mental health experiences as part of a mental health programme to help others, books may begin to value and drawn on these experiences to achieve personal goals such as greater social engagement, thus promoting social inclusion and involvement in other activities that invite them to draw on their lived expertise.

2.4 Being empowered - If living books are supported to tell their stories of mental health difficulties in a way that is authentic to their experience and aligns with their motivation for sharing their story, then they will experience a sense of empowerment because the process of storytelling will promote autonomy over expression of an experience that in society more broadly is often stigmatised and supressed.

TITLE: Designing a Library of Lived Experience for Mental Health: integrated realist synthesis and experience-based co-design study

Unintended impacts

2.5 Recalling distressing experiences - If living books tell their experience of mental health difficulties, without adequate training and support, then the process of recalling painful experiences may trigger distress because they are still emotionally processing these experiences.

2.6 Devaluing of lived experiences - If living books attempt to tell their experiences to a reader who is perceived to be disinterested, disrespectful or insensitive, then they will be at increased risk of feeling that their lived experiences of distress have devalued contributing to disengagement from the library.

3. Organisation and support

3.1 Balancing conversational openness and risk of harm - Impacts of a living library for mental health will be moderated by organisational aims, including the purpose of the library, and organisational culture, including the extent to which lived experience of distress is deemed to be an important resource for shared learning. These factors will determine how organisers, or 'librarians', understand the need to balance the facilitation of open conversations about mental health with the need to restrict or structure conversations to reduce perceived risk to participants. This will determine when and how they intervene to support conversations within the library.

3.2 Curation and commodification - If the stories selected for the library, and the way they are told (curation), is perceived by books to be prescriptive to the extent that their stories are no longer authentic expressions of their lived experience, then books may perceive that their stories of mental health experiences are being commodified – used for a purpose that is incongruent with their motivation for participating in the library - and thus devalued. This is likely to lead to them withdrawing participation in the library.

3.3 Location facilitates engagement - If the physical location in which the library is held facilitates a feeling of psychological safety, for example within a space that means personal disclosure remains private and where participants are not overlooked by the public, then both books and readers will be more likely to engage in open and engaging conversations because they will feel better able to immerse themselves in conversation without distraction or concern over confidentiality.

3.4 Perceived emotional support - Where books and readers perceive that the library provides or will provide sufficient emotional support to address any potential distress, they will feel safe and held, and so and will be more open to engaging with potentially distressing conversations about mental health.

TITLE: Designing a Library of Lived Experience for Mental Health: integrated realist synthesis and experience-based co-design study

3.5 Setting expectations - Impacts of a living library are likely to depend on the expectations of both books and readers. Where it is made clear to participants what is expected of them within the library and any boundaries in place, participants will be better equipped to manage any difficulties that may arise and/or engage with the programme as intended, because they will understand the purpose and function of the library.

3.6 Setting ground rules – Where the 'librarians' or organisers set clear guidelines for engaging in conversations about mental health, including the necessity of communicating respectfully and what to do if difficult conversation do arise, both books and readers will feel empowered to engage in more open and sincere conversation.
