# Supplementary material for: Designing a Library of Lived Experience for Mental Health: integrated realist synthesis and experience-based co-design study in UK mental health services
Source: BMJ Open. 2024 Jan 31;14(1):e081188. doi: 10.1136/bmjopen-2023-081188 (PMC10831458; doi:10.1136/bmjopen-2023-081188)
Supplement: Supplementary data [file bmjopen-2023-081188supp004.pdf]

TITLE: Designing a Library of Lived Experience for Mental Health: integrated realist synthesis and experience-based co-design study

Supplemental file 4 – search strategies

Search 1: Initial search for living library-related literature.

Searches for literature related to living libraries was intentionally broad and targeted academic and grey literature databases in health, social care, and education. An initial search retrieved articles from database inception to February 2022. A second, updated search using the same search strategy retrieved evidence from February 2022-March 2023.

Databases:

Published literature:

- Web of Science
- Scopus
- PsycINFO
- MEDLINE
- CINAHL
- Embase
- SocINDEX
- Social Care Online
- British Education Index
- Education Abstracts
- Library, Information Science & Technology Abstracts

Grey literature:

- ProQuest
- British Library
- International Clinical Trials Registry Platform
- Overton.io
- Bielefeld Academic Search Engine
- Google.com (top 100 search results)
- Scholar.google.com (top 100 search results)

Search terms:

"living librar*" OR "human librar*" applied to 'title', 'abstract', and 'keywords' fields (if available)

Eligibility criteria

- Published (including academic articles and book chapters) or grey literature (including theses, blogs, reports, conference documents and policy documents)
- Available in English
- 2000 (inception of the programme) to date
- Specifically relates to a human or living library event
- Text only

TITLE: Designing a Library of Lived Experience for Mental Health: integrated realist synthesis and experience-based co-design study

Search 2: Targeted search for theories related to psychological safety.

This search was conducted in June 2022 on Web of Science. Resources available for the review precluded full review of the research literature and as such, a large multi-disciplinary database was selected for this search. Its aim was to identify English language articles in health-related research that included middle range theories related to the concept of psychological safety. Search terms were informed by the BeHEMoTh framework [24] and combined with the 'AND' Boolean operator:

Behaviour of interest: "psychological safety"

Health context: "health"

Exclusions: n/a

Models or **th**eories: "model* or theor* or concept* or framework*"

The search returned 290 articles. Title and abstracts were screened by PM. Articles that appeared relevant to the research question and suggested that they may include a middle range theory were flagged for full-text screening. Twenty-nine articles were read by PM and RJ who held collaborative discussions, through which one article was identified as including a middle range theory of psychological safety relevant to the developing programme theory.
