# Supplementary material for: Designing a Library of Lived Experience for Mental Health: integrated realist synthesis and experience-based co-design study in UK mental health services
Source: BMJ Open. 2024 Jan 31;14(1):e081188. doi: 10.1136/bmjopen-2023-081188 (PMC10831458; doi:10.1136/bmjopen-2023-081188)
Supplement: Supplementary data [file bmjopen-2023-081188supp005.pdf]

TITLE: Designing a Library of Lived Experience for Mental Health: integrated realist synthesis and experience-based co-design study

Supplemental file 5 - Characteristics of included studies

Author(s), year	Document type	Setting, Country	Aim/objective	Book focus (General/focused)	Readers sample
Ashmore (2010)	Journal article	n/a, Canada	'…provides a discussion of the development and implementation of living library programs in the context of creating and fostering community engagement in the library.'	n/a	n/a
Bagci & Blazhenkova (2020)	Journal article	University, Turkey	'…tested the effectiveness of HL as a direct intergroup contact intervention on affective (trust, empathy), cognitive (knowledge), and behavioural (e.g., willingness to talk to outgroup members) aspects of outgroup attitudes'	General. Books included 'Schizophrenic'	University students
Blizzard, Becker, &Goebel, 2018	Journal article	University, Canada	'…describe the Augustana human library and how the team has used the event to take a new approach to teaching information literacy.'	Focused. Books related to Women's Studies	University students
Chung, Tse, & Tasha, 2022	Journal article	Educational setting, Hong Kong	'This study aimed to test the effectiveness of a human library intervention at enhancing MHL [mental health literacy]'	Focused. Books on mental health (bipolar disorder and schizophrenia)	Young adults in tertiary education
Constable & Harris, 2008	Journal article	Public libraries, UK	'… to explore a community development model of Living Library within public libraries in England.'	General	Public
Copenhaver & Koclanes, 2019	Journal article	University	'Raise awareness of social justice issues and to expose students to issues present in our local community'	General	University staff/students and the public
Dreher & Mowbray	Research monograph	Public and third sector organisations, Australia	'…comprehensive and independent analysis of Human Libraries (formerly Living Libraries) in Australia'	Varied. Typically General	Varied. Typically public.
Fortune & Leighton, 2022	Journal article	Community support centre for people with mental illness	'we report on in this paper focused on the experiences of individuals living with mental illness who became living books for an initiative aimed at addressing stigma by dispelling stereotypes related to mental illness through inclusionary dialogue.'	Focused. Mental health	Under-graduate therapeutic recreation students
Gamtso, Mannon, & Whipple, 2017	Book chapter	High school, university and public libraries, USA	'describe the planning and implementation of the New Hampshire program…and a discussion of the New Hampshire Human Library's impact on participants' learning.	Varied. Typically General	Students and/or the public

TITLE: Designing a Library of Lived Experience for Mental Health: integrated realist synthesis and experience-based co-design study

Garbutt, 2008	Conference paper	n/a, Australia	'The purpose of this paper is to begin this theoretical discussion of living libraries and their aims.	n/a	n/a
Giesler, 2021	Journal Article	University, USA	…explored two research questions: How do those in the role of enlisted participants (Books) describe the HL [human library] experience? and What potential does the HL have to create IGD [intergroup dialogue] in the social work classroom?'	General	Social work students
Giesler 2020	Magazine article	University, USA	Discusses 'What makes the Human Library so powerful? How can social work professionals and educators use it to weave together the profession's core values?'	General	University students
Giesler & Juarez, 2019	Journal Article	University, USA	'(1) to assess the power of the Living Library experience to bring people from disparate backgrounds …(2) to discern how the Living Library project builds community…(3) to add to the literature outlining potential benefits and risks of veterans who tell their stories of military service.	Focused. Books on veterans' experiences	Varied
Groyecka et al., 2019	Journal Article	Public library, Poland	…examined the effectiveness of the Human Library (held Wroclaw, Poland) in reducing social distance towards Roma, Muslims, dark-skinned, and transgender people, as well as in decreasing homonegativity.	General. Books included titles related to bulimia and anorexia, and schizophrenia.	Public
Hewlin-Via et al., 2021	Research poster	University, Australia	'To evaluate the changes in students' cultural awareness and sensitivity after attending the HL. To understand the perspective of student OTs [Occupational Therapists] in participating in the HL [Human Library] …'	General. Books included growing up with mental illness	Student occupation al therapists
Huang & Dobreski, 2017	Conference paper	Public and university libraries, USA	'We present how libraries have attempted to utilize technologies and leverage community support to enable human library events.'	General	Students and/or the public
Inkster et al., 2016	Research report	Hospital, UK	'to challenge prejudice in healthcare'.	General. Books included 'mental health problems'	Healthcare staff
Kudo et al., 2011	Conference paper	University, Japan	'…provides a first-person narrative of preparation, challenges and success of a one-day Human Library administered by 30 undergraduate students and their supervisor at a Japanese university.'	General	Public
Kwan, 2020	Journal article	Mental health service centre, Hong Kong	'explores the relevance and usefulness of this approach [living library] in a mental health setting'	Focused. Books on mental health	People in recovery and other community members

TITLE: Designing a Library of Lived Experience for Mental Health: integrated realist synthesis and experience-based co-design study

Lam, Wong & Zhang, 2023	Journal article	n/a	'…his article aims to review the current body of knowledge on HL by conducting a systematic narrative review on the existing literature.'	Varied	Varied.
Little et al., 2011	Implementa tion guide	Various, Council of Europe	'This guide is intended to provide support and direction to Living Library organisers in Europe.'	General	Varied. Typically public.
Michalak et al., 2019	Journal article	Online, Canada	'… (1) to create an innovative Web-based Bipolar Wellness Centre, (2) to conduct a mixed-methods (i.e., quantitative and qualitative) evaluation to assess the impact of different sorts of engagement (i.e., knowledge translation [KT]), and (3) to support engagement with the self-management information in the Bipolar Wellness Centre'	Focused. Books were people with experience of bipolar disorder.	People with bipolar disorder
Orosz et al., 2016	Journal article	High School, Hungary	'assesses the effectiveness of the Living Library intervention and tests whether and how perceived peer prejudices moderate its effectiveness in reducing prejudice toward Roma and LGBT people'	Focused. Books were Roma and members of the LGBT community.	High school students
Pardasani & Rivera, 2017	Thesis	University, Finland	'to serve as a guidebook in itself as a reference for the implementation guidelines, training and promotional material and a resource material for the future Human Library events at DIAK or any other social work institutions.'	General. Books included people with anxiety and depression.	Students
Pratchett, 2019	Blog	Hospital, UK	'to bring staff from different areas of the trust together and enable them to hear the lived experience of colleagues in their own words.'	General.	Healthcare staff
Russell & Linsley, 2011	Journal article	University, UK	'The project centred on the running of a 'human library' event in which adult nursing and social work students engaged with mental health service users through an open forum'.	Focused. Mental health service users	Student nurses and social workers
Sen et al., 2016	Journal article	University, UK	'describes the running of four Living Libraries on a UK postgraduate social work course'	General & focused, with Books related to the experience of social care.	Student social workers
Sen et al., 2021	Book chapter	Universities, UK & Finland	'Two approaches are presented from two universities … In the University of Sheffield (UK), people with experiences of social work services, teachers and students have developed living libraries as a regular feature of the syllabus for a qualifying master's degree programme in social work since 2014. In the Diaconia University of Applied Sciences (Diak, Finland), bachelor-level students in social services initiated and organised a Human Library event…'	General & focused, with stories related young people's experiences of social care services	Student social workers/Pu blic

TITLE: Designing a Library of Lived Experience for Mental Health: integrated realist synthesis and experience-based co-design study

Watson 2015	Thesis	University, Australia	'The research question is: What does an examination of Human Library inform us about how people can challenge prejudice and increase respect for difference as a means for promoting humans' rights and freedoms?'	General	Public
