# Supplementary material for: Designing a Library of Lived Experience for Mental Health: integrated realist synthesis and experience-based co-design study in UK mental health services
Source: BMJ Open. 2024 Jan 31;14(1):e081188. doi: 10.1136/bmjopen-2023-081188 (PMC10831458; doi:10.1136/bmjopen-2023-081188)
Supplement: Supplementary data [file bmjopen-2023-081188supp006.pdf]

TITLE: Designing a Library of Lived Experience for Mental Health: integrated realist synthesis and experience-based co-design study

Supplemental file 6 – Participant demographics

ID	Age group	Gender identity	Ethnic group	Current or previous mental health experience
1	51-60	Female	White/White British	Carer; voluntary sector staff
2	31-40	Male	Black/African/Caribbean/ Black British	Mental health service user
3	51-60	Female	White/White British	Mental health service user; carer; voluntary sector service user; voluntary sector staff
4	41-50	Male	White/White British	Mental health service user; mental health service staff; carer; voluntary sector staff
5	61-70	Male	White/White British	Mental health service user; voluntary sector staff
6	18-30	Female	Asian/Asian British	Mental health service user; carer; voluntary sector service user
7	51-60	Male	White/White British	Mental health service user
8	41-50	Male	White/White British	Mental health service staff
9	31-40	Female	White/White British	Mental health service user; mental health service staff
10	18-30	Female	White/White British	Carer; voluntary sector staff
11	51-60	Male	White/White British	Mental health service user; mental health service staff; carer
12	71-80	Male	White/White British	Mental health service user
13	41-50	Male	White/White British	Mental health service user; mental health service staff
14	31-40	Female	Other ethnic group	Voluntary sector service user
15	41-50	Male	White/White British	Mental health service user
16	51-60	Male	White/White British	Carer; voluntary sector staff
17	31-40	Female	White/White British	Mental health service user; carer; mental health service staff
18	18-30	Male	Black/African/Caribbean/ Black British	Mental health service user
19	31-40	Female	White/White British	Mental health service user; mental health service staff; carer
20	31-40	Male	White/White British	Mental health service user; voluntary sector staff
21	41-50	Female	Asian/Asian British	Carer
22	61-70	Male	White/White British	Mental health service user; voluntary sector staff
23	41-50	Female	White/White British	Carer
24	41-50	Female	Mixed/multiple ethnic groups	Mental health service user
25	61-70	Female	White/White British	Mental health service user; voluntary sector service user
26	41-50	Male	White/White British	Mental health service user
27	51-60	Female	White/White British	Mental health service user; carer; voluntary sector service user
28	18-30	Female	White/White British	Mental health service user
29	31-40	Male	Other ethnic group	Mental health service user; voluntary sector service user
30	51-60	Female	Other ethnic group	Mental health service user

TITLE: Designing a Library of Lived Experience for Mental Health: integrated realist synthesis and experience-based co-design study

31	51-60	Female	White/White British	Mental health service staff; carer
