# Supplementary material for: Designing a Library of Lived Experience for Mental Health: integrated realist synthesis and experience-based co-design study in UK mental health services
Source: BMJ Open. 2024 Jan 31;14(1):e081188. doi: 10.1136/bmjopen-2023-081188 (PMC10831458; doi:10.1136/bmjopen-2023-081188)
Supplement: Author's manuscript [file bmjopen-2023-081188.draft_revisions.pdf]

BMJ Open

BMJ Open is committed to open peer review. As part of this commitment we make the peer review history of every article we publish publicly available.

When an article is published we post the peer reviewers' comments and the authors' responses online. We also post the versions of the paper that were used during peer review. These are the versions that the peer review comments apply to.

The versions of the paper that follow are the versions that were submitted during the peer review process. They are not the versions of record or the final published versions. They should not be cited or distributed as the published version of this manuscript.

BMJ Open is an open access journal and the full, final, typeset and author-corrected version of record of the manuscript is available on our site with no access controls, subscription charges or pay-per-view fees (http://bmjopen.bmj.com).

If you have any questions on BMJ Open's open peer review process please email info.bmjopen@bmj.com

BMJ Open

Designing a Library of Lived Experience for Mental Health: integrated realist synthesis and experience-based co-design study

Journal:	*BMJ Open*
Manuscript ID	bmjopen-2023-081188
Article Type:	Original research
Date Submitted by the Author:	23-Oct-2023

Complete List of Authors:	Marshall, Paul; Lancaster University, Spectrum Centre for Mental Health Research, Division of Health Research<br>Lobban, Fiona; Lancaster University, Spectrum Centre for Mental Health Research, Division of Health Research<br>Barbrook, John; Lancaster University, Library<br>Collins, Grace; Freelance participatory artist<br>Foster, Sheena; Lancaster University, Spectrum Centre for Mental Health Research, Division of Health Research<br>Glossop, Zoe; Lancaster University, Spectrum Centre for Mental Health Research, Division of Health Research<br>Inkster, Claire; Health Education England North West<br>Jebb, Paul; Lancashire and South Cumbria NHS Foundation Trust, Patient Experience, Engagement & Safeguarding<br>Johnston, Rose; Lancaster University, Spectrum Centre for Mental Health Research, Division of Health Research<br>Khan, Hameed; Lancaster University, Spectrum Centre for Mental Health Research, Division of Health Research<br>Lodge, Christopher; Lancaster University, Spectrum Centre for Mental Health Research, Division of Health Research<br>Machin, Karen; Independent survivor researcher<br>Michalak, Erin; The University of British Columbia, Department of Psychiatry<br>Powell, Sarah; Lancaster University, Lancaster Medical School<br>Russell, Samantha; Lancaster University, Spectrum Centre for Mental Health Research, Division of Health Research<br>Rycroft-Malone, Jo; Lancaster University, Faculty of Health and Medicine<br>Slade, Mike; University of Nottingham School of Health Sciences; Nord University, Faculty of Medicine and Health Sciences<br>Whittaker, Lesley; Lancashire and South Cumbria NHS Foundation Trust, Patient Experience, Engagement & Safeguarding<br>Jones, Steven; Lancaster University, Spectrum Centre for Mental Health Research, Division of Health Research

Keywords:	QUALITATIVE RESEARCH, Social Support, MENTAL HEALTH

For peer review only - http://bmjopen.bmj.com/site/about/guidelines.xhtml

I, the Submitting Author has the right to grant and does grant on behalf of all authors of the Work (as defined in the below author licence), an exclusive licence and/or a non-exclusive licence for contributions from authors who are: i) UK Crown employees; ii) where BMJ has agreed a CC-BY licence shall apply, and/or iii) in accordance with the terms applicable for US Federal Government officers or employees acting as part of their official duties; on a worldwide, perpetual, irrevocable, royalty-free basis to BMJ Publishing Group Ltd ("BMJ") its licensees and where the relevant Journal is co-owned by BMJ to the co-owners of the Journal, to publish the Work in this journal and any other BMJ products and to exploit all rights, as set out in our licence.

The Submitting Author accepts and understands that any supply made under these terms is made by BMJ to the Submitting Author unless you are acting as an employee on behalf of your employer or a postgraduate student of an affiliated institution which is paying any applicable article publishing charge ("APC") for Open Access articles. Where the Submitting Author wishes to make the Work available on an Open Access basis (and intends to pay the relevant APC), the terms of reuse of such Open Access shall be governed by a Creative Commons licence – details of these licences and which Creative Commons licence will apply to this Work are set out in our licence referred to above.

Other than as permitted in any relevant BMJ Author's Self Archiving Policies, I confirm this Work has not been accepted for publication elsewhere, is not being considered for publication elsewhere and does not duplicate material already published. I confirm all authors consent to publication of this Work and authorise the granting of this licence.

For peer review only

TITLE: Designing a Library of Lived Experience for Mental Health: integrated realist synthesis and experience-based co-design study

Word Count: 3980

Authors and affiliations

Corresponding author: Paul Marshall, Spectrum Centre for Mental Health Research, Lancaster University, Lancaster, UK, p.marshall4@lancaster.ac.uk

Fiona Lobban, Spectrum Centre for Mental Health Research, Lancaster University, Lancaster, UK

John Barbrook, Lancaster University Library, Lancaster, UK

Grace Collins, freelance participatory artist, UK

Sheena Foster, Spectrum Centre for Mental Health Research, Lancaster University, Lancaster, UK

Zoe Glossop, Spectrum Centre for Mental Health Research, Lancaster University, Lancaster, UK

Clare Inkster, NHS England North West, Manchester, UK

Paul Jebb, Patient Experience, Engagement & Safeguarding, Lancashire and South Cumbria NHS Foundation Trust, Preston

Rose Johnston, Spectrum Centre for Mental Health Research, Lancaster University, Lancaster, UK

Hameed Khan, Spectrum Centre for Mental Health Research, Lancaster University, Lancaster, UK

Christopher Lodge, Spectrum Centre for Mental Health Research, Lancaster University, Lancaster, UK

Karen Machin, independent survivor researcher, UK

Erin E. Michalak, Department of Psychiatry, University of British Columbia, Vancouver, British Columbia, Canada

Sarah Powell, Lancaster Medical School, Lancaster University, Lancaster, UK

Samantha Russell, Spectrum Centre for Mental Health Research, Lancaster University, Lancaster, UK

Jo Rycroft-Malone, Faculty of Health and Medicine, Lancaster University, Lancaster, UK

Mike Slade, School of Health Sciences, Institute of Mental Health, University of Nottingham, Nottingham, UK; Faculty of Medicine and Health Sciences, Nord University, Namsos, Norway

Lesley Whittaker, Patient Experience, Engagement & Safeguarding, Lancashire and South Cumbria NHS Foundation Trust, Preston, UK

Steven Jones, Spectrum Centre for Mental Health Research, Lancaster University, Lancaster UK

For peer review only

ABSTRACT

Objective

Living Library 'Readers' can learn about experiences of others through conversations with living 'Books'. This study sought to generate a realist programme theory and a theory-informed implementation guide for a Library of Lived Experience for Mental Health (LoLEM).

Design

Integrated realist synthesis and experience-based co-design.

Setting

Ten online workshops with participants based in the North of England.

Participants

Thirty-one participants with a combination of personal experience of using mental health services, caring for someone with mental health difficulties, and/or working in mental health support roles.

Results

Database searches identified 30 published and grey literature evidence sources which were integrated with data from 10 online co-design workshops. The analysis generated a programme theory comprising five context-mechanism-outcome (CMO) configurations. For Readers, direct conversations humanise others' experiences (CMO 2) and provide the opportunity to flexibly explore new ways of living (CMO 3). Through participation in a Living Library, Books may experience personal empowerment (CMO 4), while the process of self-authoring and co-editing their story (CMO 5) can contribute to personal development. This programme theory informed the co-design of an implementation guide highlighting the importance of tailoring event design and participant support to the contexts in which LoLEM events are held.

Conclusions

The LoLEM has appeal across stakeholder groups and can be applied flexibly in a range of mental health-related settings. Implementation and evaluation are required to better understand the positive and negative impacts on Books and Readers.

Registration

PROSPERO CRD42022312789

Strengths and limitations of this study

- This study used a novel, iterative, and creative approach to integrating theory development and intervention co-design.
- The programme theory provides a conceptual basis for further evaluation of a LoLEM, including outcomes for those participating in events as 'Books' and 'Readers'.
- This study informed detailed, co-designed implementation guidance for use by LoLEM organisers.
- However, the LoLEM is yet to be delivered as a sustained programme of events, so long-term impacts require further investigation.

Keywords

For peer review only

Mental Health Services, Psychosocial Intervention, Qualitative Research, Social Support

For peer review only

INTRODUCTION

The value of sharing health related experiences is widely recognised. Varied contexts draw on these experiences, including for shaping research [1], enriching professional education [2], and informing peer support [3]. There is an expanding evidence-base focused specifically on mental health lived experiences. For example, social contact interventions reduce mental health stigma [4], mental health peer support contributes to improvements in psychosocial outcomes [5], and there is growing interest in the use of mental health narratives to achieve a range of organisational aims [6]. The importance of integrating lived experience perspectives in health service development is acknowledged in UK and global policy [7,8], emphasising the need for continued efforts to promote lived experience perspectives.

Existing models for sharing mental health experiences do have limitations. Many opportunities are offered only periodically by health services and educational institutions, such as those linked to service improvement [9] or health professional training [10]. Others require commitment to more formal positions, including the peer-support worker role in the UK [11], which may preclude access by those with existing commitments. There is an outstanding need to extend such opportunities to groups whose voices are often not heard in mainstream conversations about mental health, including those from marginalised communities [12].

Adaptable approaches are required for expanding opportunities to share and learn from mental health experiences. The Living Library may represent one such model. Pioneered by the Human Library Organisation [13], it draws on a library metaphor to invite people, or 'Readers', to learn directly from living 'Books', who are supported to discuss important aspects of their life experience in short conversations [14]. Events are facilitated by staff, or 'Librarians', who provide guidance to those involved. These events are often held in open settings, including public and university libraries, and have typically aimed to challenge a range of societal prejudices by facilitating interactions between people who may not otherwise engage in conversation [15]. The model has also been used in a mental health context to address stigma [16] and facilitate peer-support [17]. However, implementation recommendations sensitive to the specific challenges of discussing mental health experiences are limited.

Following UK complex intervention development guidance calling for greater emphasis on theory development and stakeholder engagement [18], we applied a novel integration of realist synthesis and experience-based co-design (EBCD) to explore the Living Library as a strategy for sharing mental health experiences. Realist synthesis is a method of evidence synthesis used to develop causal statements, or programme theories, to explain how social programmes work, for whom and under which circumstances [19]. EBCD identifies opportunities for healthcare innovation and draws on stakeholder perspectives to generate creative solutions [20]. Our integrative approach is detailed in a published research protocol [21].

Objective

- To use realist synthesis to develop a programme theory for a mental health-focused Living Library, which we term a Library of Lived Experience for Mental Health (LoLEM).

- To use insights from this synthesis to inform co-design workshops with a range of mental health stakeholders, with the goal of developing an accessible implementation guide.

METHOD

This study received ethical approval from Coventry and Warwick National Health Service Research Ethics Committee (ref: 305975). The systematic search strategy was developed with an information specialist (JB) and pre-registered on PROSPERO https://www.crd.york.ac.uk/prospero/display_record.php?RecordID=312789. RAMESES guidance informed the reporting of this study [22].

As described in the study protocol [21], theory development and EBCD workstreams were iterative and ran in parallel. However, the study broadly progressed through the following stages:

Eliciting initial theories and touchpoints

Initial programme theories (IPTs) were elicited through theory gleaning interviews [23] with 6 members of an expert advisory group and the study public and patient involvement (PPI) lead, all of whom had been involved in participating in or researching Living Libraries in the domains of mental health (EM, MS, SF, CL), education (SF) or stigma reduction (CI, HK) (topic guide available in supplemental file 1). Twenty IPTs were refined through advisory group discussion (supplemental file 2). IPTs highlighted key issues to be explored with participants in co-design workshops, referred to as 'touchpoints' in the EBCD process [20].

Retrieving relevant evidence

Searches were conducted on research databases and grey literature sources (figure 1) from 2000, the inception of the initial Human Library approach [13], to March 2023. Preparatory unsystematic searches indicated that relatively few articles in this literature were likely to focus solely on mental health. Therefore, reports on Living Libraries related to specific topics including mental health and from generalist events featuring a range of lived experiences were included. Following title and abstract screening, the full-texts of identified articles were judged for inclusion using a realist-informed assessment of relevance and rigour (PM) with 20% independently assessed by a second reviewer (RJ). Citation chaining was used to identify articles in reference sections and 'cited by' pages of articles included in the initial search. IPTs and initial workshops highlighted psychological safety as a key theoretical and implementation focus for this study. Consistent with the iterative nature of realist synthesis searches [24], we conducted a targeted search of a large multidisciplinary database for existing formal theories related to the concept and included an additional evidence source [25]. A full description of each search is available in supplemental file 3.

Iterating initial theories

IPTs were added to NVivo 12 as nodes to create an initial theory framework [26]. PM conducted coding of the full text of each evidence source. This involved identifying and labelling sections of text related to potential contextual factors, mechanisms, and/or outcomes, as defined in realist methodology [19]. Codes were linked to the most relevant initial programme theory node. PM, FL, and RJ refined the initial theory framework by reformulating, consolidating, and combining initial statements based on whether underlying data supported, refuted, or refined individual IPTs. While reviewing this evidence, retroductive reasoning was used to hypothesise causal forces behind regularities apparent in the data. An iterative process of written feedback and group discussion led to the consolidation of the initial framework into a series of candidate CMO configurations.

Establishing a multi-stakeholder co-design group

For peer review only

Eligible EBCD participants were adults with any self-identified mental health experience. We recruited broadly through local mental health services, health research networks, and third sector organisations primarily based in the North of England. Workshops were facilitated by KM and GC, both experts in mental health peer-support who deliver workshops from a lived experience perspective. Workshops were supported by researchers (ZG, RJ, PM), senior nurses (PJ, LW), a service user researcher (CL), and academic clinical psychologist (SJ).

Designing workshops reflexively

We ran 10, 2-hour online co-design workshops over 12 months. Workshops were flexibly designed in team meetings around two primary goals. First, we aimed to develop a comprehensive implementation guide. This required the team to reflect on gaps in knowledge and plan further exploration of topics pertinent to implementation. Second, we explored 'touchpoints' to facilitate programme theory refinement. One such example was the exploration of psychological safety in workshop 4, a key concept in the IPT framework which required further investigation to understand relevant implantation factors.

Developing and refining outputs

Workshop participants' views and implementation suggestions were captured using the collaborative online note taking platform Jamboard [27] and in researcher (CL, PM, RJ, SJ, ZG) field notes. Data were combined in workshop summary documents following each session, reviewed by the research team in post-workshop de-briefs, and added to NVivo for integration into the ongoing analysis.

Summary documents informed an initial draft implementation guide, refined through feedback from the EBCD group. Implementation recommendations from the co-design process were linked to the developing theoretical framework. CMOs were further refined through discussion and written feedback from the wider research team and expert advisory group.

Final versions of the LoLEM programme theory and implementation guide were shared during an interactive dissemination event at which the LoLEM was piloted and the guide made freely available [28].

RESULTS

Results of systematic searches

Database and grey literature searches returned 30 eligible evidence sources. Figure 1 describes the process of evidence identification. Study characteristics are available in supplemental file 4.

Figure 1. Flow diagram of evidence identification

[figure 1 here]

For peer review only

Description of co-design workshops

EBCD workshops were attended by 31 participants, 16 of whom identified as female and 15 male. Workshops were attended by people with experience of using mental health services (n=23), informal caregiving (n=13), and working in mental health services (n=8) or in voluntary sector mental health support roles (n=8). Participant demographic details are available in supplemental file 5.

Details of activities completed at each workshop are presented in Table 1.

Table 1. Experience-based co-design workshop details

Workshop	n	Activities
1	25	• Project introduction<br>• Story formulation activity
2	27	• Story sharing activity<br>• Reflection on personal boundaries
3	25	• Creating a fictional Reader 'persona'<br>• Reflection on Library design for different Reader groups
4	24	• What influences potential Readers' decisions to participate?<br>• What constitutes a safe space for sharing mental health experiences?<br>• How can event organisers cultivate psychological safety amongst Books and Readers?
5	25	• Reflection on participants' preferred roles at a Living Library (Books, Readers, or Librarians)<br>• Planning a hypothetical Living Library<br>• A 'design to fail' activity, considering factors likely to contribute to unsuccessful events
6	23	• Design considerations for Living Libraries in different contexts (the voluntary, the NHS, and public libraries)
7	23	• Identifying resources required to run successful Living Libraries<br>• A 'sales pitch' activity to potential funders
8	21	• Advantages and disadvantages of delivering Living Libraries with different aims (mental health support versus changing public perception of mental health)<br>• Co-design of the implementation guide contents page
9	20	• The desired characteristics of event staff ('Librarians')<br>• Considering how to evaluate a Living Library<br>• Feedback on the implementation guide
10	17	• Celebration event<br>• Review of project successes and thanking participants for their contributions

LoLEM programme theory

We developed a programme theory comprising five CMO configurations, with corresponding implementation recommendations (Figure 2).

Figure 2. LoLEM programme theory with implementation recommendations

[figure 2 here]

CMO 1 – Psychological safety facilitates dialogue

When event organisers implement guidance and support strategies tailored for their organisational settings (context), participants will be better able to understand the Living Library model and how to manage personal boundaries such that they feel in control of their experience (mechanism). This will promote participants' perceptions of psychological safety (outcome) necessary for engaging and productive conversations.

Psychological safety refers to an individual's comfort with taking interpersonal risks to meet their goals within a given organisational context [25]. The Living Library model provides a platform for participants to take interpersonal risks by sharing experiences in direct conversation. We propose that psychological safety creates a facilitating context, or 'ripple' effect [29], promoting the likelihood of subsequent positive impacts described by CMOs 2-5.

Developing psychological safety for Books and Readers commonly involves implementing event rules to explicitly promote mutual respect, awareness of boundaries, and a sense of personal control. Pre-event briefings may be used to reinforce the principles of the approach: "'Readers are required to return the 'Book' in the same psychological and physical condition and are asked: 'Never harm a Book!'" [30]. Promoting psychological safety for Books can involve offering training and practice sessions to set expectations, identify what they wish to share, and prepare for questions they may not want to answer. EBCD participants suggested that psychological safety would further be promoted by the reassuring presence of attentive staff, or 'Librarians', able to provide guidance, oversight, and emotional support where necessary.

Given their conversational focus, Living Libraries hold potential for Books and Readers to experience interpersonal challenges that could undermine psychological safety. Books may be exposed to negative attitudes towards mental health, feel pressure to be an exhaustive authority on the issues they discuss, 'pumped' for knowledge by repeated readings, or alternatively, 'left on the shelf' by uninterested Readers [31,32]. Readers themselves may experience discomfort hearing directly about others' distress. The emergent nature of these interactions means that such circumstances cannot be entirely predicted, yet event planners should prepare for and mitigate these eventualities as far as possible by foregrounding implementation strategies (table 3) to promote psychological safety.

CMO 2 – Humanising others' experiences

Direct conversations with Books (context) humanise the experience of mental health difficulties (mechanism), contributing to Readers developing a greater empathetic understanding of mental health (outcome).

Event organisers framed direct interaction between Books and Readers as an explicit goal. Many aimed "to bring people together who may not otherwise meet and to also kind of shut down the stereotypes that people have about others" [33]. Events focused on challenging prejudice sought to provide a platform for Books to "show the general public they are 'human'" [32] and challenge superficial views of important aspects of their experiences and identities by breaking down

For peer review only

perceived “us-them” divides between Books and Readers [34]. This could occur through recognition of shared experience including emotional difficulties, to which Readers could relate regardless of personal differences: “Even women [who don’t] look like me, or identify as the same race as me still go through similar struggles. So that was eye-opening because I’ve never really thought about it like that” [35].

This more nuanced understanding of others’ experiences was for some facilitated by the subtle emotional and behavioural cues present in direct interaction, which augmented the interpersonal connection shared by Books and Readers [33,36]. In this context, the experiences and identities Books represent shift from abstract and disembodied concepts to personified and meaningful human experiences [37,38]. Mental health difficulties are thus re-conceptualised from “myths to storied realities” [42], enhancing Readers’ abilities to subsequently understand and empathise with the perspectives of people experiencing similar difficulties [34].

CMO 3 – Exploring new ways of living

When Living Libraries provide Readers with the permission to explore Books’ experiences through synchronous interaction (context), they will use this opportunity to flexibly explore issues of personal significance (mechanism). This will facilitate awareness of new and helpful ways of living (outcomes).

The synchronous nature of Living Library conversations allows Readers to personalise their interactions by asking questions about aspects of Books’ stories that resonate with their own. As noted by a participant in a mental health-specific event [34], “in a talk, listeners listen passively. As speakers, we are asked to tell our whole story. But in the Human Library, it does not matter whether the sharing is complete or not. The important thing is to let readers know what they want to know.” This form of experiential learning can influence how Readers understand ways of managing their own distress [17] and may help health professionals identify new ways of supporting service users [37,39].

EBCD participants noted that the relative novelty of the Living Library approach and the associated ‘Book’ metaphor can imply that the sharing of experience is intended to be unidirectional, with Readers taking a passive role. Organisers can facilitate interactive dialogue by providing Readers with explicit permission to explore questions of personal significance, within established boundaries. Strategies for reaffirming Readers’ conversational permission include providing library attendees with clear ground rules and example questions or cues to prompt engagement.

CMO 4 – Personal empowerment

When Living Libraries facilitate the authentic expression of Books’ personal experiences (context), Books will feel that their expertise has been heard and valued (mechanism), contributing to personal empowerment (outcome).

Living Libraries have been used to spotlight marginalised voices [40]. The approach both promotes Books’ sense of having been heard as experts by experience and provides a medium to use their stories to meet personally valued goals, including shaping public attitudes and offering support to others. Personal empowerment therefore emerges from this opportunity for direct self-representation, recognition, and the pursuit of positive change [31,41]. EBCD group members highlighted the potential of a LoLEM to meet the motivation of many experts by experience in

mental health to use their unique perspectives to inspire individual, organisational, and social progress against a wider context of stigma and under-recognition. This was mirrored in literature describing participants' sense of pride and satisfaction after sharing their stories at Living Library events [38,42,43]:

The EBCD group also identified how personal empowerment can occur through meaningful participation in event design and delivery. Extensive involvement of experts by experience was suggested to diminish power imbalances between those with lived experience and organisations, such as health services and universities, that may host a LoLEM. Workshops highlighted that the greater the degree of lived experience involvement and collaborative working alongside staff, the more likely a LoLEM is to reflect the perspective of the groups it seeks to engage. It was suggested that this may reduce the potential for disempowerment emerging from peoples' stories being misused.

CMO 5 – Self authoring and co-editing

When Living Libraries support Books with developing a personal narrative to share in conversation with Readers (context), Books will explore and develop insight into these aspects of their lives (mechanism). This leads Books to develop new ways of understanding and sharing their experiences which can contribute to personal growth (outcome).

For Books, authoring and articulating a personal narrative represents a "self-directed process of discovery" [31] and ongoing engagement with story sharing can "demonstrate how their personal identities evolve and develop over time" [43]. This process may contribute to the reframing of mental health difficulties as an aspect of past experience that is of value to the present self [42]. Relatively unstructured dialogue may also facilitate a form of narrative co-editing: "…in the process of creating a narrative in cooperation with readers, books actually alter their understanding of their own self-appointed topic and what it means to them" [31]. Personal growth can therefore result from a shift in the Books' perceptions of their current circumstances through reflexive story refinement: "Despite occasional moments of discomfort and, perhaps in some cases, because of them, Books recognized that their stories changed because of their participation in the HL [Human Library] Project. Details were added, elements they believed were less important emerged as such, and overall, they achieved greater clarity about their narratives" [35].

Table 2 provides illustrative data supporting each CMO.

Table 2 Programme theories with illustrative data extracts

For peer review only

Programme theory	Illustrative data	Source
1. Psychological safety facilitates dialogue	"[Books'] needs must be recognised and met. This could include things like limiting the number of readings a living book can be required to participate in within a given session, providing adequate support for living books including those with special needs, and providing time and structure for living books to debrief after readings or to read other living books"	[31]
	" [a living library conversation] will be a deep dive, might need to help these first-time Books prepare e.g. with practice and storytelling and thinking about boundaries"	Workshop 2 Jamboard
2. Humanising others' experiences	"Readers were given permission to empathize with the experiences of Books and, in the process, see themselves in the Books' stories"	[35]
	"Stories trigger a response in us that helps us empathise with others"	Workshop 3 field notes
3. Exploring new ways of living	"Another Reader found inspiration in a Book's journey that he could apply to his own life: "I learned from some people's experiences that, okay, I'm going through this right now, but you went through it and you overcame it. So I have a choice."	[41]
	"[Books are] providing other visions/real life examples of ways forward that are hopeful"	Workshop 8 Jamboard
4. Personal empowerment	"Living libraries excel as a strategy for giving voice to marginalised groups. Living library conversations allow for direct self-representation, unmediated by third parties."	[40]
	"Mental health has been stigmatised for so long, people ignored, locked away and forgotten. Just the very action of telling someone that you want to hear their story is massive."	Workshop 2 Jamboard
5. Self-authoring and co-editing	"By thinking about and structuring their story, people will often 're-author' their lives, by defining their own existence in relationship to themselves and what they were going through at the time; thereby constructing reality by choices made to give meaning to their lives"	[44]
	"Storytelling can be therapeutic for teller"	Workshop 2 Jamboard

Implementation guidance

The integrated realist synthesis and EBCD process contributed to the development of a theory informed implementation guide. Recommendations are summarised here with reference to key stages of event delivery (Table 3). The full guide is available online [28].

For peer review only

Table 3. Co-designed implementation recommendations by event delivery stages

	Set-up
Team building (CMO1, CMO4)	Ensure the event team includes people with the skills to plan, promote, and implement a mental health-related event. Embed the perspectives of experts by experience, including in the mechanism by which stories are selected. Recruit books with diverse relevant experiences. Where applicable, provide context-appropriate and timely payment.
Goal setting (CMO1)	Identify a clear goal for the event. Align this with plans for recruitment, support provision, and promotion.
Venue selection (CMO1)	Select a non-stigmatising venue that provides a degree of privacy for individual discussions yet does not leave participants feeling isolated and exposed in conversations with people they may not have met before. Conduct accessibility and safeguarding assessments.
	Pre-event
Book training (CMO5)	Provide comprehensive training to Books. Focus on helping Books to understand the model, what is expected of them, and where to go for support. Clearly identify personal boundaries for story sharing and how to manage them. Generate a synopses and story title. Consider developing individualised support plans.
Team practice (CMO1, CMO5)	Rehearse how the Living Library will be delivered. Books may wish to practice sharing their stories with team members and/or each other to refine their story and prepare for the event.
Develop materials (CMO4)	Develop event materials alongside experts by experience. This may include guidance for readers highlighting what the event is for, rules for participation, and support materials.
	At the Library
Ground-rules (CMO1, CMO2, CMO3)	Clearly outline ground-rules for conversations. This may include instructions for engaging in respectful dialogue and for Books and Readers to be mindful of any boundaries for their conversations, such as to avoid certain topics. Encourage readers to explore topics and questions of personal interest within these boundaries.
Content Warnings (CMO1)	If appropriate, consider generating content warnings which could be included in Books' synopses.
Attentive support (CMO1)	Ensure that emotional support is consistent with the nature and scale of the event. Develop plans for managing distress, including risk-related scenarios.
	Post-event
De-briefing (CMO1)	Implement individual or group debriefs. Consider providing Readers with the opportunity to reflect on their experiences, for example in a quite space away from the main event.
Signposting (CMO1)	If appropriate, make available relevant and up-to-date sign-posting material.

For peer review only

Supervision (CMO1, CMO5)	Consider ongoing supervision for Books and Librarians. This could include a review of any difficulties, support needs, or changes to Books' stories. Where available, discuss further opportunities for lived-experience involvement within the organisation.

DISCUSSION

This integrated realist synthesis and EBCD study generated a programme theory for a LoLEM and theory-informed implementation recommendations. Results emphasise the importance of psychological safety for facilitating productive conversations between Books and Readers. Implementation recommendations highlight ways in which potential organisers may seek to foster supported environments for sharing mental health experiences and further suggestions are reported in a co-designed implementation guide [28].

This study builds on implementation recommendations for the Human Library Organisation's generalist approach [14]. A notable difference in the context of mental health is the centrality of psychological safety, defined as the ability to take desired interpersonal risks. Occupational research indicates that psychological safety is associated with proactive communication including concern-raising within teams, individual and team-level learning, and work engagement [25]. Recommendations for promoting psychological safety within this literature suggest organisations should seek to create cultures defined by collaboration and interpersonal openness [45]. This aligns with established principles-based approaches to mental health peer-support [46], which point to the significance of safe and trusting relationships within organisations that draw on lived experience, and control over how those experiences are shared. Results here suggest that by investing in training and building meaningful relationships with those sharing their stories, LoLEM organisers can clearly articulate expectations and promote informed personal disclosure. Drawing on established measures [25], further research could evaluate the extent to which these practices influence Books' perceptions of psychological safety.

Values underpinning mental health peer support, such as mutuality and reciprocity [47], are reflected in evidence of positive impacts amongst those delivering and accessing these services. For example, benefits for those receiving support include improvements in social functioning, hope, and stigma reduction, while peer supporters may experience greater confidence, skills development, and positive reframing of personal identity [48]. Building on this evidence, results of this study suggest that a LoLEM could provide opportunities to learn about ways of understanding and managing mental health from people who may be empowered by being supported to share their stories. Indeed, participants in a Living Library focused on bipolar self-management welcomed the opportunity for direct contact with peers [17], demonstrating the feasibility of using this approach in the peer support context. A LoLEM could also assist service users with sharing stories as part of a recovery-oriented programme of support [34]. Indeed, there is an established body of literature describing the use of storytelling in mental health [6,49] indicating that developing a personal narrative can be highly meaningful for the teller, yet also potentially emotionally challenging [50]. This reinforces this study's emphasis on dedicated support with story formulation for potential Books.

The LoLEM approach requires adaptation for specific contexts. Organisers should consider factors that could undermine the safe and empowering implementation of lived-experience focused programmes. Survivor research identifies how power imbalance between those with lived

experience and health services could serve to discourage perspectives deemed unacceptable to institutional agendas, including critical and marginalised voices, or delegitimize those who draw on their experience in pursuit of systemic change [51]. A recent systematic review of lived experience narratives drew attention to potential misuses of mental health experiences, including commodification, coerced elicitation, harm to narrators or audiences, and curatorial decisions that limit diversity [6]. Good practice recommendations relevant to the LoLEM model include proactive reflection on organisers' personal biases to ensure power imbalances are not reinforced, encouraging audiences to endorse ethical listening and openness to difference, seeking consent, and promoting control over personal narratives [6]. Consistent with this, findings here emphasise the vital role of lived experience perspectives in implementing transparent and collaborative LoLEMs. Established models for involvement, including EBCD, could be used to facilitate development of future programmes.

Implications for research

Further work may explore the feasibility of Living Libraries as sustained programmes and whether effects persist over time. CMOs reported here could inform the selection of outcomes. The humanising impact of a LoLEM would be expected to contribute to more empathetic perspectives on mental health and in the context of peer-support, future research may investigate potential impacts on Reader self-efficacy. For Books, the proposed positive impact of storytelling and personal empowerment aligns with definitions of personal recovery for which several assessments have been developed [52]. Factors underpinning differences in outcomes across varied service settings, and how this model may fit alongside existing lived experienced-based interventions, are currently unknown. These issues could be addressed using realist evaluation methodology.

Implications for practice

While lived experience opportunities continue to grow within health services, most roles do not involve training in self-disclosure [53]. The LoLEM model could bridge the gap between people with mental health experiences seeking opportunities to help others, and more formal paid positions requiring considerable time commitments. The approach could also complement efforts to increase lived experience expertise in health professional training, as evidenced by its successful implementation in social work training [37]. More specifically, a LoLEM could afford healthcare trainees opportunities to engage with a broader range of people than they may typically meet, and in neutral settings outside of clinical environments which may be more conducive to open conversation. It is also feasible to combine a Living Library with structured reflexive activities to embed learning and consider the applicability of a Book's story to a Reader's clinical practice [39]. Actions to promote equality, inclusion and diversity in the UK health service should apply an intersectional lens [54]. It has been noted that the Living Library model is well suited to assist health professional trainees with developing a lived appreciation of intersectionality through conversations that illuminate how aspects of identity interact [35]. As per the programme theory reported here a LoLEM could serve to humanise and promote the understanding of nuanced relationships between mental health experiences and other salient aspects of identity.

Strengths and limitations

A strength of this approach is the breadth of mental health experience represented in the study team. PPI perspectives were sought throughout, including during study design, via an advisory group and in co-design meetings, and to assist with dissemination. This facilitated the generation of findings that reflect a variety of perspectives and contexts in which a LoLEM could be hosted. This

For peer review only

project also demonstrates the feasibility of a novel and creative approach to mental health programme co-design and theory generation. However, this study does have limitations. Many evidence sources identified in systematic searches were non-mental health specific and involved short-term evaluations. This study principally recruited from the North of England which may limit the transferability of its findings. In a change to the study protocol [21], theory refinement interviews [23] were not conducted due to limitations in available resources. The reported search on psychological safety was similarly limited to a single multidisciplinary database due to a limited capacity to manage the screening burden of a larger search.

CONCLUSION

This project is the first to apply realist methods to articulate explanations for how Living Libraries may work and adds to existing literature by identifying specific strategies for promoting the safety and effectiveness of this model as applied to mental health experiences. Continued recognition of the importance of lived experience in shaping mental health policy and practice justifies further consideration of how this approach can be implemented and evaluated.

FUNDING

This work was funded by the National Institute for Health and Care Research (NIHR) under its Research for Patient Benefit (RfPB) Programme (Grant Reference Number NIHR203476). The views expressed are those of the author(s) and not necessarily those of the NIHR or the Department of Health and Social Care. The study was sponsored by Lancashire and South Cumbria NHS Foundation Trust.

COMPETING INTEREST STATEMENT

None declared.

PUBLIC AND PATIENT INVOLVEMENT

Experts by experience in mental health contributed to all stages of this study. A stakeholder group informed the initial study design and experts by experience were co-applicants for funding. The study team included a service user research and experts by experience were involved in an advisory group, all of whom are involved in authoring key study outputs. The co-design group involved service users and carers and was led by facilitators who delivered workshops from a lived experience perspective. Participants and team members with lived experience assisted with dissemination at local and national events.

ETHICS APPROVAL

This study involved human participants and granted ethical approval by Coventry and Warwick National Health Service Research Ethics Committee on 29th December 2021 (REF: 305975). Participants gave informed consent to participate in the study before taking part.

DATA AVAILABILITY STATEMENT

No data are available.

AUTHOR CONTRIBUTIONS

For peer review only

FL designed and led the study. PM conducted data collection and analysis for the realist synthesis. JB supported search strategy development. KM and GC co-facilitated EBCD workshops with support from SJ, RJ, ZG, CL, PJ and LW. JR-M provided methodological guidance for the realist synthesis. SF, CI, HK, SP, EM, and MS formed an expert advisory group which provided strategic guidance across the study. SR supported article writing. All authors provided approved the final article.

ORCID IDs

Mike Slade 0000000170203434

Steven Jones 0000-0002-8801-5113

Samantha J Russell 0000-0003-1904-9739

Paul Marshall 0000-0002-3913-3359

Sarah Powell 0000-0002-3509-5789

Zoe Glossop 0000-0002-6151-4860

Fiona Lobban 0000-0001-6594-4350

TWITTER

Steven Jones @lancsspectrum

Sarah Powell @DrSarahPowell1

John Barbrook @stackingbooks

Paul Jebb @pauljebb1

Lesley Whittaker @Lesleyp222

ACKNOWLEDGMENTS

The research team would like to thank all those who contributed to the co-design of the outputs for this study.

For peer review only

REFERENCES

1 Capobianco L, Faija C, Cooper B, *et al.* A framework for implementing Patient and Public Involvement in mental health research: The PATHWAY research programme benchmarked against NIHR standards. *Health Expect* 2023.

2 Rowland P, Anderson M, Kumagai AK, *et al.* Patient involvement in health professionals' education: A meta-narrative review. *Adv Health Sci Educ* 2019;**24**:595–617.

3 Shalaby RAH, Agyapong VI. Peer support in mental health: literature review. *JMIR Ment Health* 2020;**7**:e15572.

4 Maunder RD, White FA. Intergroup contact and mental health stigma: A comparative effectiveness meta-analysis. *Clin Psychol Rev* 2019;**72**:101749.

5 White S, Foster R, Marks J, *et al.* The effectiveness of one-to-one peer support in mental health services: a systematic review and meta-analysis. *BMC Psychiatry* 2020;**20**:1–20.

6 Yeo C, Rennick-Egglestone S, Armstrong V, *et al.* Uses and misuses of recorded mental health lived experience narratives in healthcare and community settings: systematic review. *Schizophr Bull* 2022;**48**:134–144.

7 National Health Service. The NHS Long Term Plan. 2019. https://www.longtermplan.nhs.uk/wp-content/uploads/2019/08/nhs-long-term-plan-version-1.2.pdf

8 World Health Organization. Comprehensive mental health action plan 2013–2030. 2021.

9 Veldmeijer L, Terlouw G, Van Os J, *et al.* The Involvement of Service Users and People With Lived Experience in Mental Health Care Innovation Through Design: Systematic Review. *JMIR Ment Health* 2023;**10**:e46590.

10 Happell B, Byrne L, McAllister M, *et al.* Consumer involvement in the tertiary-level education of mental health professionals: A systematic review. *Int J Ment Health Nurs* 2014;**23**:3–16.

11 Beales A, Wilson J. Peer support–the what, why, who, how and now. *J Ment Health Train Educ Pract* 2015;**10**:314–324.

12 Gillard S. Guest editorial: re-balancing the discourse, inviting critique and welcoming marginalised voices in research about peer support in mental health services. Adv. Ment. Health. 2022;**20**:87–90.

13 Human Library Organisation. About the Human Library. https://humanlibrary.org/about/

14 Little N, Nemutlu G, Magic J, *et al.* Don't judge a book by its cover! *Living Libr Organiser's Guide* 2011.

For peer review only

15 Lam GYH, Wong HT, Zhang M. A Systematic Narrative Review of Implementation, Processes, and Outcomes of Human Library. *Int J Environ Res Public Health* 2023;**20**.

16 Chung EY-H, Tse TT-O. Effect of human library intervention on mental health literacy: a multigroup pretest-posttest study. *BMC Psychiatry* 2022;**22**:73.

17 Michalak EE, Morton E, Barnes SJ, *et al.* Supporting Self-Management in Bipolar Disorder: Mixed-Methods Knowledge Translation Study. *JMIR Ment Health* 2019;**6**:e13493.

18 Skivington K, Matthews L, Simpson SA, *et al.* A new framework for developing and evaluating complex interventions: update of Medical Research Council guidance. *bmj* 2021;**374**.

19 Hunter R, Gorely T, Beattie M, *et al.* Realist review. *Int Rev Sport Exerc Psychol* 2022;**15**:242–265.

20 Green T, Bonner A, Teleni L, *et al.* Use and reporting of experience-based codesign studies in the healthcare setting: a systematic review. *BMJ Qual Saf* 2020;**29**:64–76.

21 Lobban F, Marshall P, Barbrook J, *et al.* Designing a library of lived experience for mental health (LoLEM): protocol for integrating a realist synthesis and experience based codesign approach. *BMJ Open* 2023;**13**:e068548.

22 Wong G, Greenhalgh T, Westhorp G, *et al.* RAMESES publication standards: realist syntheses. *BMC Med* 2013;**11**:1–14.

23 Mukumbang FC, Marchal B, Van Belle S, *et al.* Using the realist interview approach to maintain theoretical awareness in realist studies. *Qual Res* 2020;**20**:485–515.

24 Booth A, Carroll C. Systematic searching for theory to inform systematic reviews: is it feasible? Is it desirable? *Health Inf Libr J* 2015;**32**:220–235.

25 Newman A, Donohue R, Eva N. Psychological safety: A systematic review of the literature. *Hum Resour Manag Rev* 2017;**27**:521–535.

26 Gilmore B, McAuliffe E, Power J, *et al.* Data analysis and synthesis within a realist evaluation: toward more transparent methodological approaches. *Int J Qual Methods* 2019;**18**:1609406919859754.

27 Google. Jamboard. https://jamboard.google.com/

28 Marshall P, Jones S, Lodge C, *et al. Library of Lived Experience for Mental Health Implementation Guide*. 2023. https://doi.org/10.5281/zenodo.7920240

29 Jagosh J, Bush PL, Salsberg J, *et al.* A realist evaluation of community-based participatory research: partnership synergy, trust building and related ripple effects. *BMC Public Health* 2015;**15**:1–11.

30 Bagci SC, Blazhenkova O. Unjudge Someone: Human Library as a Tool to Reduce Prejudice toward Stigmatized Group Members. *Basic Appl Soc Psychol* 2020;**42**:413–31.

31 Ashmore A. Alive with knowledge: Engaging communities through living libraries. *Libr Stud J* 2010;**5**:14–14.

For peer review only

32 Dreher T, Mowbray J. The power of one on one: Human libraries and the challenges of antiracism work. 2012.

33 Huang Y, Dobreski B, Xia H. Human library: Understanding experience sharing for community knowledge building. 2017;:1152–65.

34 Kwan CK. A Qualitative Inquiry into the Human Library Approach: Facilitating Social Inclusion and Promoting Recovery. *Int J Environ Res Public Health* 2020;**17**.

35 Giesler MA. Humanizing Oppression: The Value of the Human Library Experience in Social Work Education. *J Soc Work Educ* Published Online First: 2021.

36 Blizzard K, Becker Y, Goebel N. Bringing Women's Studies to Life: Integrating a Human Library into Augustana's Women's Studies Curriculum. *Coll Q* 2018;**21**:n3.

37 Sen R, McClelland N, Jowett B. Belonging to the library: humanising the space for social work education. *Soc Work Educ* 2016;**35**:892–904.

38 Sen R, Nylund M, Ali Hayward R, *et al.* The Living Library in social work education. *Involv Serv Users Soc Work Educ Res Policy Comp Eur Anal* 2021;:73.

39 Inkster M C; St Jean, L; Elliott, P; Leach. Challenging Prejudice in Healthcare – the Human Library. 2016. https://www.nwpgmd.nhs.uk/sites/default/files/The%20Human%20Library%20for%20Healthcare%20Learners%20%282%29.pdf

40 Garbutt R. The living library: some theoretical approaches to a strategy for activating human rights and peace. *Act Hum Rights Peace Univers Responsib Conf 2008 Conf Proc Aust* 2008;:270–8.

41 Giesler M. Human Libraries. *New Soc Work* 2020;**27**:24–8.

42 Fortune D, Leighton J. Mental illness as a valued identity: How a leisure initiative promoting connection and understanding sets the stage for inclusion. *Leisure Sciences (2022): 1-21.*

43 Watson GJ. "You shouldn't have to suffer for being who you are": An Examination of the Human Library Strategy for Challenging Prejudice and Increasing Respect for Difference. 2015.

44 Russell E, Linsley P. Human libraries: removing stigma. *Br J Wellbeing* 2011;**2**:15–7.

45 Edmondson AC, Lei Z. Psychological safety: The history, renaissance, and future of an interpersonal construct. *Annu Rev Organ Psychol Organ Behav* 2014;**1**:23–43.

46 Gillard S, Foster R, Gibson S, *et al.* Describing a principles-based approach to developing and evaluating peer worker roles as peer support moves into mainstream mental health services. *Ment Health Soc Incl* 2017;**21**:133–143.

47 Repper J, Aldridge B, Gilfoyle S, *et al. Peer support workers: Theory and practice*. Centre for Mental Health. London. 2013.

48 Shalaby RAH, Agyapong VI. Peer support in mental health: literature review. *JMIR Ment Health* 2020;**7**:e15572.

For peer review only

49 De Vecchi N, Kenny A, Dickson-Swift V, *et al.* How digital storytelling is used in mental health: A scoping review. *Int J Ment Health Nurs* 2016;**25**:183–193.

50 Nurser KP, Rushworth I, Shakespeare T, *et al.* Personal storytelling in mental health recovery. *Ment Health Rev J* 2018;**23**:25–36.

51 Kalocsai C, Agrawal S, de Bie L, *et al.* Power to the people? A co-produced critical review of service user involvement in mental health professions education. *Adv Health Sci Educ* 2023;:1–28.

52 Van Weeghel J, van Zelst C, Boertien D, *et al.* Conceptualizations, assessments, and implications of personal recovery in mental illness: A scoping review of systematic reviews and meta-analyses. *Psychiatr Rehabil J* 2019;**42**:169.

53 Opie JE, McLean SA, Vuong AT, *et al.* Training of Lived Experience Workforces: A Rapid Review of Content and Outcomes. *Adm Policy Ment Health Ment Health Serv Res* 2023;**50**:177–211.

54 NHS. NHS equality, diversity, and inclusion improvement plan. https://www.england.nhs.uk/long-read/nhs-equality-diversity-and-inclusion-improvement-plan/#interventions-by-protected-characteristic

For peer review only

TITLE: Designing a Library of Lived Experience for Mental Health: integrated realist synthesis and experience-based co-design study

Figure 1. Flow diagram of evidence identification

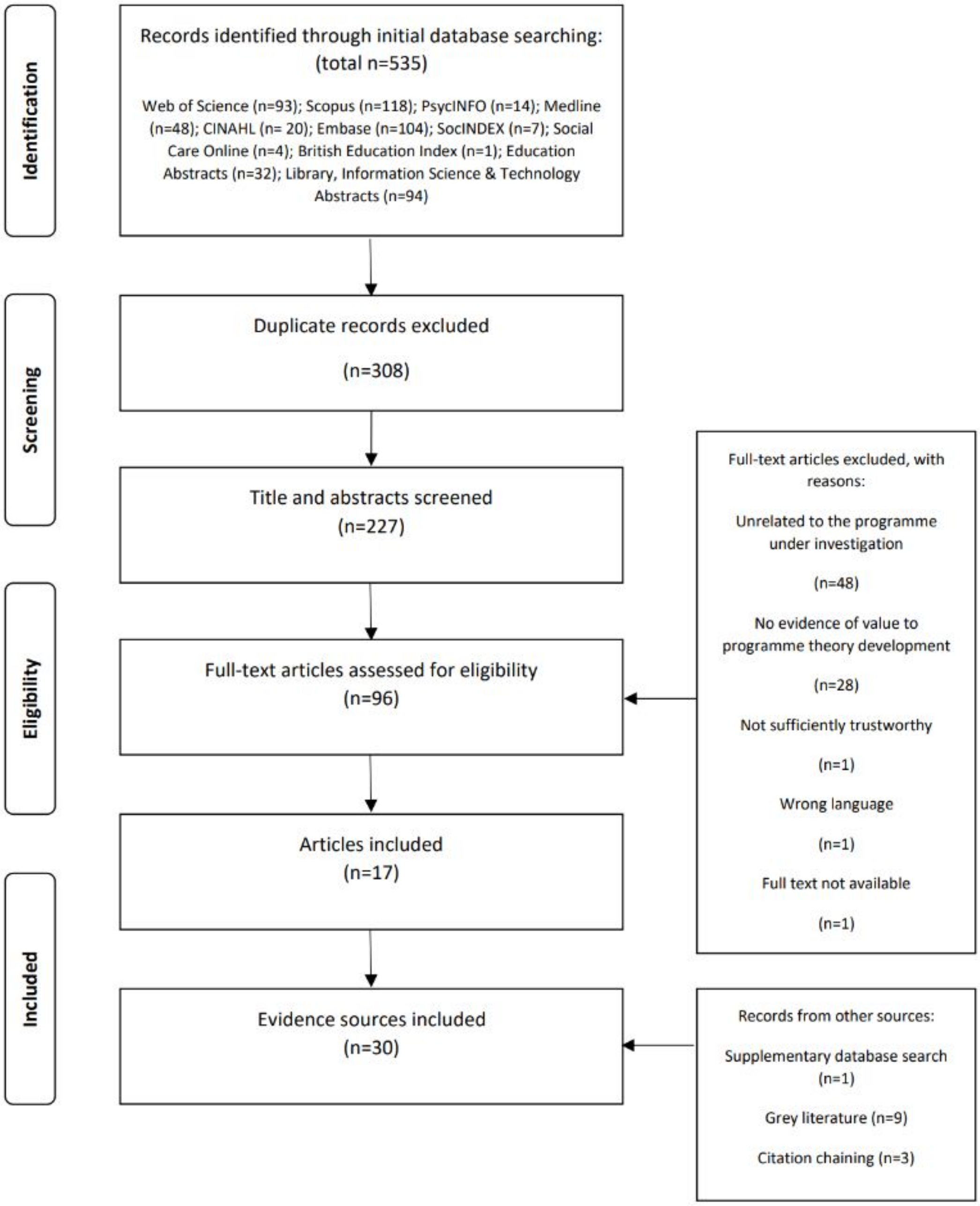

TITLE: Designing a Library of Lived Experience for Mental Health: integrated realist synthesis and experience-based co-design study

Figure 2. LoLEM programme theory with implementation recommendations

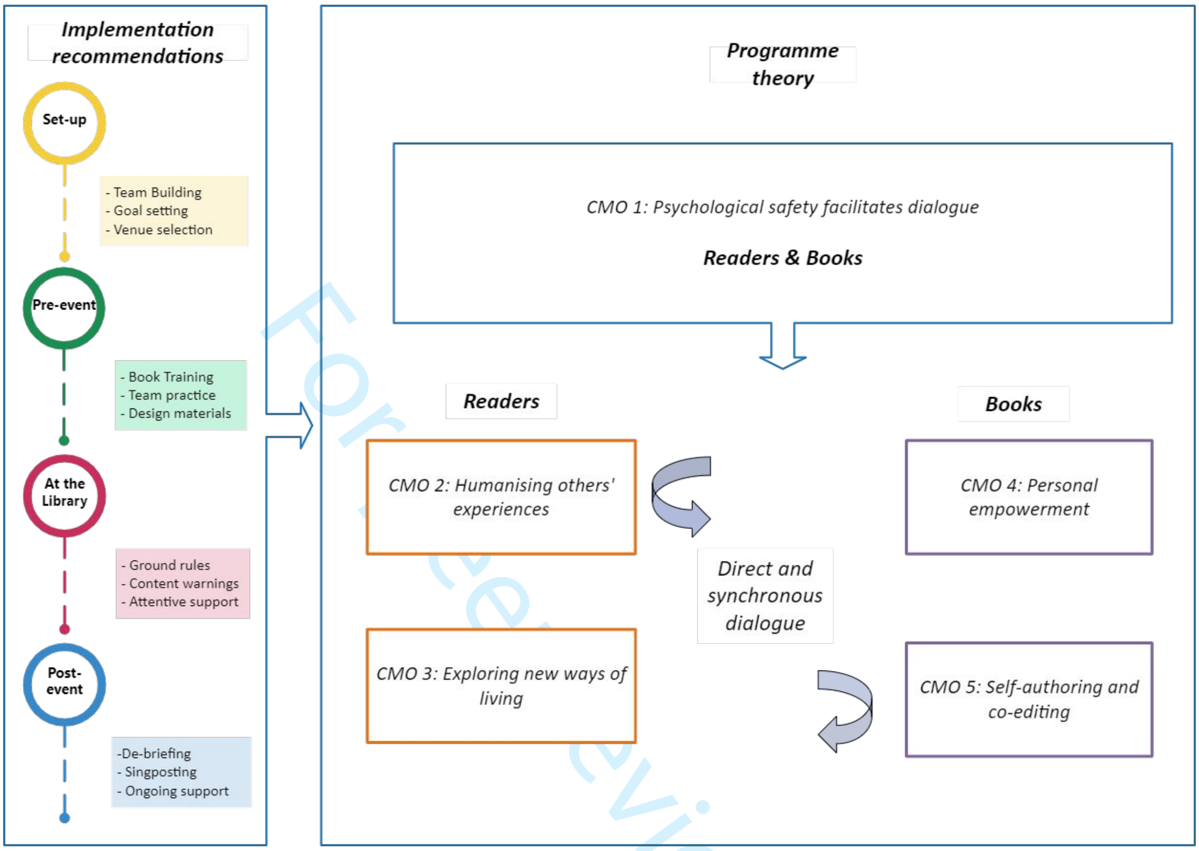

For peer review only

TITLE: Designing a Library of Lived Experience for Mental Health: integrated realist synthesis and experience-based co-design study

Supplemental file 1 – Interview topic guide

The topic guide should be amended to reflect the interviewees' experiences of living libraries as books/readers/organisers/researchers. In section 1, focus on developing an understanding of participant experiences by using open ended, exploratory questioning. In section 2, if applicable, use the developing programme theory to further explore participants' experiences with reference to the specific hypotheses being tested at that stage of theory development.

Section 1: Participant experiences of living libraries

Suggested topics and example questions (select as appropriate). These topics remain flexible as per the semi-structured interview method.

Topic 1: Type of event and context

- Did you attend as a book/reader/organiser?
- What was the purpose of the living library event you attended/organised?
- How did you find out/how were you recruited/how did you recruit for the event?
- What was the format of the event? In-person/online? Time restricted conversations? One off or recurring events?
- Were you given instructions prior to the event/did you provide training for attendees?

Topic 2: Sharing experiences

For peer review only

TITLE: Designing a Library of Lived Experience for Mental Health: integrated realist synthesis and experience-based co-design study

- Did you have any views/expectations about sharing/hearing a story before the event?
- How did you find out about the books that were available to speak with/provide options for readers?
- How did you decide who to speak to/when?
- What was the format of the discussions?

Topic 3: Impacts

- How did you experience sharing your story/hearing someone else's story?
- What was it like speaking with someone about [the topic] in particular?
- Were there unexpected effects of taking part?
- What effect (if any) did the living library event on your views of the topics discussed, and why?

Section 2: Theory testing

Following the guidance for realist interviews provided by Manzano (2016), prior to each interview, consider how the current programme theory can be further understood with reference to context, mechanism and outcome. Adapt the interview questions depending on the stage of theory development: in early interviews make greater use of general and exploratory questions to inform tentative theories; later, theory refinement focused interviews should increase in specificity and may draw on specific examples/cases to illustrate CMOs in discussions with participants; theory consolidation focuses on refining existing hypotheses, for example, how CMOs may vary depending on different stakeholder perspectives and how CMOs may link together across the programme theory.

For peer review only

TITLE: Designing a Library of Lived Experience for Mental Health: integrated realist synthesis and experience-based co-design study

Note: examples of how CMOs may be developed with a focus on theory development (adapted from Manzano, 2016):

Exploring context:

What characteristics of the living library location contribute to people feeling able to share their stories?

Looking for mechanisms:

How do you think the living library event influenced the attitudes of those who took part?

Looking for intended and unintended outcomes:

In your opination, how should participants be supported when asked to share their experiences as part of a living library?

References

Manzano, A. (2016). The craft of interviewing in realist evaluation. *Evaluation*, *22*(3), 342-360.

For peer review only

TITLE: Designing a Library of Lived Experience for Mental Health: integrated realist synthesis and experience-based co-design study

Supplemental file 2 - Initial programme theories

1. Readers – hearing stories about mental health

Intended impacts

1.1 Reduced isolation - If readers hear stories that resonate with their own experiences of mental health difficulties, then they will feel that their experiences are shared, thus normalising their experience and reducing their sense of social isolation. This shared experience may make them feel they are more likely to be understood by the book, and by others with similar experiences, and therefore more able to talk about their mental health difficulties with others, promoting social engagement and help-seeking.

1.2 Tailored learning - If readers take part in a library that facilitates synchronous interaction, then they will be able to ask the questions about mental health that they want answered, increasing the relevance of the information shared by the book to their personal experience and individual learning needs. This will mean readers are better informed and have the information they need to manage their own mental health difficulties.

1.3 Challenging preconceptions - If readers interact with a living book who through their participation in the library challenges the reader's negative preconceptions or stigma regarding mental health, for example that it is not possible to live well with a mental health problem, then contact with the book will disconfirm these preconceptions, increasing hope for their own recovery.

1.4 Connecting to the individual - If the reader engages with a book in a synchronous, face-to-face interaction, then this intimate interpersonal context facilitated will promote empathy with the book as their storytelling will be perceived as more authentic and emotive, facilitating other intended impacts (such as learning, social connection, and hope for recovery).

1.5 Connecting to the story - If the reader interacts with a book whose story involves a compelling narrative about their mental health difficulties, then the reader will be more engaged by their story, making the story more likely to be internalised and remembered, facilitating other impacts such as lasting change in perspective. This impact will be greater in a context where shared life experiences create personal resonance with the story.

Unintended impacts

For peer review only

TITLE: Designing a Library of Lived Experience for Mental Health: integrated realist synthesis and experience-based co-design study

1.6 Exposure to distressing stories - If the reader interacts with a book who recalls distressing aspects of their experiences and/or who becomes distressed when doing so, then the reader may be at increased risk of themselves becoming distressed.

1.7 Interpersonal tension - If the reader engages with a book who they perceive holds views that they find objectionable, then there is an increased risk of interpersonal tension and possible subsequent distress is increased The reader is also unlikely to identify with the book, and more likely to actively reject any strategies or ideas that the book offers, so will not learn anything of value and feel they have wasted their time. This could make them less likely to engage with other forms of peer-based support in future.

1.8 Negative social comparison - If the reader engages in negative social comparison, for example perceives that they are unlikely to achieve the level of recovery experienced by the living books at the library, then they may feel dejected and less hopeful about their own recovery, and therefore less likely to engage in recovery focussed activities. This is particularly likely to happen if the books are selected for “success” (or “recovery”) stories only.

2. Living ‘books’ – telling stories about mental health

Intended impacts

2.1 Using experiences to help others - If living books tell their stories of mental health difficulties to readers who they feel have been derived benefit from hearing their experiences, those telling their stories will derive satisfaction from their participation contributing to a sense of personal meaning.

2.2 Developing personal understanding - If living books tell their stories of mental health difficulties, then the process of recalling and communicating their story in a safe environment may facilitate the development of new understandings of their previous experiences, contributing to greater awareness of their own mental health.

2.3 Social inclusion - Where living books are encouraged to use their mental health experiences as part of a mental health programme to help others, books may begin to value and drawn on these experiences to achieve personal goals such as greater social engagement, thus promoting social inclusion and involvement in other activities that invite them to draw on their lived expertise.

2.4 Being empowered - If living books are supported to tell their stories of mental health difficulties in a way that is authentic to their experience and aligns with their motivation for sharing their story, then they will experience a sense of empowerment because the process of storytelling will promote autonomy over expression of an experience that in society more broadly is often stigmatised and supressed.

For peer review only

TITLE: Designing a Library of Lived Experience for Mental Health: integrated realist synthesis and experience-based co-design study

Unintended impacts

2.5 Recalling distressing experiences - If living books tell their experience of mental health difficulties, without adequate training and support, then the process of recalling painful experiences may trigger distress because they are still emotionally processing these experiences.

2.6 Devaluing of lived experiences - If living books attempt to tell their experiences to a reader who is perceived to be disinterested, disrespectful or insensitive, then they will be at increased risk of feeling that their lived experiences of distress have devalued contributing to disengagement from the library.

3. Organisation and support

3.1 Balancing conversational openness and risk of harm - Impacts of a living library for mental health will be moderated by organisational aims, including the purpose of the library, and organisational culture, including the extent to which lived experience of distress is deemed to be an important resource for shared learning. These factors will determine how organisers, or 'librarians', understand the need to balance the facilitation of open conversations about mental health with the need to restrict or structure conversations to reduce perceived risk to participants. This will determine when and how they intervene to support conversations within the library.

3.2 Curation and commodification - If the stories selected for the library, and the way they are told (curation), is perceived by books to be prescriptive to the extent that their stories are no longer authentic expressions of their lived experience, then books may perceive that their stories of mental health experiences are being commodified – used for a purpose that is incongruent with their motivation for participating in the library - and thus devalued. This is likely to lead to them withdrawing participation in the library.

3.3 Location facilitates engagement - If the physical location in which the library is held facilitates a feeling of psychological safety, for example within a space that means personal disclosure remains private and where participants are not overlooked by the public, then both books and readers will be more likely to engage in open and engaging conversations because they will feel better able to immerse themselves in conversation without distraction or concern over confidentiality.

3.4 Perceived emotional support - Where books and readers perceive that the library provides or will provide sufficient emotional support to address any potential distress, they will feel safe and held, and so and will be more open to engaging with potentially distressing conversations about mental health.

For peer review only

TITLE: Designing a Library of Lived Experience for Mental Health: integrated realist synthesis and experience-based co-design study

3.5 Setting expectations - Impacts of a living library are likely to depend on the expectations of both books and readers. Where it is made clear to participants what is expected of them within the library and any boundaries in place, participants will be better equipped to manage any difficulties that may arise and/or engage with the programme as intended, because they will understand the purpose and function of the library.

3.6 Setting ground rules – Where the 'librarians' or organisers set clear guidelines for engaging in conversations about mental health, including the necessity of communicating respectfully and what to do if difficult conversation do arise, both books and readers will feel empowered to engage in more open and sincere conversation.

For peer review only

TITLE: Designing a Library of Lived Experience for Mental Health: integrated realist synthesis and experience-based co-design study

Supplemental file 3 – search strategies

Search 1: Initial search for living library-related literature.

Searches for literature related to living libraries was intentionally broad and targeted academic and grey literature databases in health, social care, and education. An initial search retrieved articles from database inception to February 2022. A second, updated search using the same search strategy retrieved evidence from February 2022-March 2023.

Databases:

Published literature:

- Web of Science
- Scopus
- PsycINFO
- MEDLINE
- CINAHL
- Embase
- SocINDEX
- Social Care Online
- British Education Index
- Education Abstracts
- Library, Information Science & Technology Abstracts

Grey literature:

- ProQuest
- British Library
- International Clinical Trials Registry Platform
- Overton.io
- Bielefeld Academic Search Engine
- Google.com (top 100 search results)
- Scholar.google.com (top 100 search results)

Search terms:

"living librar*" OR "human librar*" applied to 'title', 'abstract', and 'keywords' fields (if available)

Eligibility criteria

- Published (including academic articles and book chapters) or grey literature (including theses, blogs, reports, conference documents and policy documents)
- Available in English
- 2000 (inception of the programme) to date
- Specifically relates to a human or living library event
- Text only

For peer review only

TITLE: Designing a Library of Lived Experience for Mental Health: integrated realist synthesis and experience-based co-design study

Search 2: Targeted search for theories related to psychological safety.

This search was conducted in June 2022 on Web of Science. Resources available for the review precluded full review of the research literature and as such, a large multi-disciplinary database was selected for this search. Its aim was to identify English language articles in health-related research that included middle range theories related to the concept of psychological safety. Search terms were informed by the BeHEMoTh framework [24] and combined with the 'AND' Boolean operator:

Behaviour of interest: "psychological safety"

Health context: "health"

Exclusions: n/a

Models or **th**eories: "model* or theor* or concept* or framework*"

The search returned 290 articles. Title and abstracts were screened by PM. Articles that appeared relevant to the research question and suggested that they may include a middle range theory were flagged for full-text screening. Twenty-nine articles were read by PM and RJ who held collaborative discussions, through which one article was identified as including a middle range theory of psychological safety relevant to the developing programme theory.

For peer review only

TITLE: Designing a Library of Lived Experience for Mental Health: integrated realist synthesis and experience-based co-design study

Supplemental file 4 - Characteristics of included studies

Author(s), year	Document type	Setting, Country	Aim/objective	Book focus (General/focused)	Readers sample
Ashmore (2010)	Journal article	n/a, Canada	'…provides a discussion of the development and implementation of living library programs in the context of creating and fostering community engagement in the library.'	n/a	n/a
Bagci & Blazhenkova (2020)	Journal article	University, Turkey	'…tested the effectiveness of HL as a direct intergroup contact intervention on affective (trust, empathy), cognitive (knowledge), and behavioural (e.g., willingness to talk to outgroup members) aspects of outgroup attitudes'	General. Books included 'Schizophrenic'	University students
Blizzard, Becker, &Goebel, 2018	Journal article	University, Canada	'…describe the Augustana human library and how the team has used the event to take a new approach to teaching information literacy.'	Focused. Books related to Women's Studies	University students
Chung, Tse, & Tasha, 2022	Journal article	Educational setting, Hong Kong	'This study aimed to test the effectiveness of a human library intervention at enhancing MHL [mental health literacy]'	Focused. Books on mental health (bipolar disorder and schizophrenia)	Young adults in tertiary education
Constable & Harris, 2008	Journal article	Public libraries, UK	'… to explore a community development model of Living Library within public libraries in England.'	General	Public
Copenhaver & Koclanes, 2019	Journal article	University	'Raise awareness of social justice issues and to expose students to issues present in our local community'	General	University staff/students and the public
Dreher & Mowbray	Research monograph	Public and third sector organisations, Australia	'…comprehensive and independent analysis of Human Libraries (formerly Living Libraries) in Australia'	Varied. Typically General	Varied. Typically public.
Fortune & Leighton, 2022	Journal article	Community support centre for people with mental illness	'we report on in this paper focused on the experiences of individuals living with mental illness who became living books for an initiative aimed at addressing stigma by dispelling stereotypes related to mental illness through inclusionary dialogue.'	Focused. Mental health	Under-graduate therapeutic recreation students
Gamtso, Mannon, & Whipple, 2017	Book chapter	High school, university and public libraries, USA	'describe the planning and implementation of the New Hampshire program…and a discussion of the New Hampshire Human Library's impact on participants' learning.	Varied. Typically General	Students and/or the public

For peer review only

TITLE: Designing a Library of Lived Experience for Mental Health: integrated realist synthesis and experience-based co-design study

Garbutt, 2008	Conference paper	n/a, Australia	'The purpose of this paper is to begin this theoretical discussion of living libraries and their aims.	n/a	n/a
Giesler, 2021	Journal Article	University, USA	…explored two research questions: How do those in the role of enlisted participants (Books) describe the HL [human library] experience? and What potential does the HL have to create IGD [intergroup dialogue] in the social work classroom?'	General	Social work students
Giesler 2020	Magazine article	University, USA	Discusses 'What makes the Human Library so powerful? How can social work professionals and educators use it to weave together the profession's core values?'	General	University students
Giesler & Juarez, 2019	Journal Article	University, USA	'(1) to assess the power of the Living Library experience to bring people from disparate backgrounds …(2) to discern how the Living Library project builds community…(3) to add to the literature outlining potential benefits and risks of veterans who tell their stories of military service.	Focused. Books on veterans' experiences	Varied
Groyecka et al., 2019	Journal Article	Public library, Poland	…examined the effectiveness of the Human Library (held Wroclaw, Poland) in reducing social distance towards Roma, Muslims, dark-skinned, and transgender people, as well as in decreasing homonegativity.	General. Books included titles related to bulimia and anorexia, and schizophrenia.	Public
Hewlin-Via et al., 2021	Research poster	University, Australia	'To evaluate the changes in students' cultural awareness and sensitivity after attending the HL. To understand the perspective of student OTs [Occupational Therapists] in participating in the HL [Human Library] …'	General. Books included growing up with mental illness	Student occupation al therapists
Huang & Dobreski, 2017	Conference paper	Public and university libraries, USA	'We present how libraries have attempted to utilize technologies and leverage community support to enable human library events.'	General	Students and/or the public
Inkster et al., 2016	Research report	Hospital, UK	'to challenge prejudice in healthcare'.	General. Books included 'mental health problems'	Healthcare staff
Kudo et al., 2011	Conference paper	University, Japan	'…provides a first-person narrative of preparation, challenges and success of a one-day Human Library administered by 30 undergraduate students and their supervisor at a Japanese university.'	General	Public
Kwan, 2020	Journal article	Mental health service centre, Hong Kong	'explores the relevance and usefulness of this approach [living library] in a mental health setting'	Focused. Books on mental health	People in recovery and other community members

For peer review only

TITLE: Designing a Library of Lived Experience for Mental Health: integrated realist synthesis and experience-based co-design study

Lam, Wong & Zhang, 2023	Journal article	n/a	'…his article aims to review the current body of knowledge on HL by conducting a systematic narrative review on the existing literature.'	Varied	Varied.
Little et al., 2011	Implementation guide	Various, Council of Europe	'This guide is intended to provide support and direction to Living Library organisers in Europe.'	General	Varied. Typically public.
Michalak et al., 2019	Journal article	Online, Canada	'… (1) to create an innovative Web-based Bipolar Wellness Centre, (2) to conduct a mixed-methods (i.e., quantitative and qualitative) evaluation to assess the impact of different sorts of engagement (i.e., knowledge translation [KT]), and (3) to support engagement with the self-management information in the Bipolar Wellness Centre'	Focused. Books were people with experience of bipolar disorder.	People with bipolar disorder
Orosz et al., 2016	Journal article	High School, Hungary	'assesses the effectiveness of the Living Library intervention and tests whether and how perceived peer prejudices moderate its effectiveness in reducing prejudice toward Roma and LGBT people'	Focused. Books were Roma and members of the LGBT community.	High school students
Pardasani & Rivera, 2017	Thesis	University, Finland	'to serve as a guidebook in itself as a reference for the implementation guidelines, training and promotional material and a resource material for the future Human Library events at DIAK or any other social work institutions.'	General. Books included people with anxiety and depression.	Students
Pratchett, 2019	Blog	Hospital, UK	'to bring staff from different areas of the trust together and enable them to hear the lived experience of colleagues in their own words.'	General.	Healthcare staff
Russell & Linsley, 2011	Journal article	University, UK	'The project centred on the running of a 'human library' event in which adult nursing and social work students engaged with mental health service users through an open forum'.	Focused. Mental health service users	Student nurses and social workers
Sen et al., 2016	Journal article	University, UK	'describes the running of four Living Libraries on a UK postgraduate social work course'	General & focused, with Books related to the experience of social care.	Student social workers
Sen et al., 2021	Book chapter	Universities, UK & Finland	'Two approaches are presented from two universities … In the University of Sheffield (UK), people with experiences of social work services, teachers and students have developed living libraries as a regular feature of the syllabus for a qualifying master's degree programme in social work since 2014. In the Diaconia University of Applied Sciences (Diak, Finland), bachelor-level students in social services initiated and organised a Human Library event…'	General & focused, with stories related young people's experiences of social care services	Student social workers/Public

For peer review only

TITLE: Designing a Library of Lived Experience for Mental Health: integrated realist synthesis and experience-based co-design study

Watson 2015	Thesis	University, Australia	'The research question is: What does an examination of Human Library inform us about how people can challenge prejudice and increase respect for difference as a means for promoting humans' rights and freedoms?'	General	Public

For peer review only

TITLE: Designing a Library of Lived Experience for Mental Health: integrated realist synthesis and experience-based co-design study

Supplemental file 5 – Participant demographics

ID	Age group	Gender identity	Ethnic group	Current or previous mental health experience
1	51-60	Female	White/White British	Carer; voluntary sector staff
2	31-40	Male	Black/African/Caribbean/ Black British	Mental health service user
3	51-60	Female	White/White British	Mental health service user; carer; voluntary sector service user; voluntary sector staff
4	41-50	Male	White/White British	Mental health service user; mental health service staff; carer; voluntary sector staff
5	61-70	Male	White/White British	Mental health service user; voluntary sector staff
6	18-30	Female	Asian/Asian British	Mental health service user; carer; voluntary sector service user
7	51-60	Male	White/White British	Mental health service user
8	41-50	Male	White/White British	Mental health service staff
9	31-40	Female	White/White British	Mental health service user; mental health service staff
10	18-30	Female	White/White British	Carer; voluntary sector staff
11	51-60	Male	White/White British	Mental health service user; mental health service staff; carer
12	71-80	Male	White/White British	Mental health service user
13	41-50	Male	White/White British	Mental health service user; mental health service staff
14	31-40	Female	Other ethnic group	Voluntary sector service user
15	41-50	Male	White/White British	Mental health service user
16	51-60	Male	White/White British	Carer; voluntary sector staff
17	31-40	Female	White/White British	Mental health service user; carer; mental health service staff
18	18-30	Male	Black/African/Caribbean/ Black British	Mental health service user
19	31-40	Female	White/White British	Mental health service user; mental health service staff; carer
20	31-40	Male	White/White British	Mental health service user; voluntary sector staff
21	41-50	Female	Asian/Asian British	Carer
22	61-70	Male	White/White British	Mental health service user; voluntary sector staff
23	41-50	Female	White/White British	Carer
24	41-50	Female	Mixed/multiple ethnic groups	Mental health service user
25	61-70	Female	White/White British	Mental health service user; voluntary sector service user
26	41-50	Male	White/White British	Mental health service user
27	51-60	Female	White/White British	Mental health service user; carer; voluntary sector service user
28	18-30	Female	White/White British	Mental health service user

For peer review only

TITLE: Designing a Library of Lived Experience for Mental Health: integrated realist synthesis and experience-based co-design study

29	31-40	Male	Other ethnic group	Mental health service user; voluntary sector service user
30	51-60	Female	Other ethnic group	Mental health service user
31	51-60	Female	White/White British	Mental health service staff; carer

For peer review only

BMJ Open

Designing a Library of Lived Experience for Mental Health: integrated realist synthesis and experience-based co-design study in UK mental health services

Journal:	*BMJ Open*
Manuscript ID	bmjopen-2023-081188.R1
Article Type:	Original research
Date Submitted by the Author:	03-Jan-2024
Complete List of Authors:	Marshall, Paul; Lancaster University, Spectrum Centre for Mental Health Research, Division of Health Research<br>Barbrook, John; Lancaster University, Library<br>Collins, Grace; Freelance participatory artist<br>Foster, Sheena; Lancaster University, Spectrum Centre for Mental Health Research, Division of Health Research<br>Glossop, Zoe; Lancaster University, Spectrum Centre for Mental Health Research, Division of Health Research<br>Inkster, Claire; Health Education England North West<br>Jebb, Paul; Lancashire and South Cumbria NHS Foundation Trust, Patient Experience, Engagement & Safeguarding<br>Johnston, Rose; Lancaster University, Spectrum Centre for Mental Health Research, Division of Health Research<br>Jones, Steven; Lancaster University, Spectrum Centre for Mental Health Research, Division of Health Research<br>Khan, Hameed; Lancaster University, Spectrum Centre for Mental Health Research, Division of Health Research<br>Lodge, Christopher; Lancaster University, Spectrum Centre for Mental Health Research, Division of Health Research<br>Machin, Karen; Independent survivor researcher<br>Michalak, Erin; The University of British Columbia, Department of Psychiatry<br>Powell, Sarah; Lancaster University, Lancaster Medical School<br>Russell, Samantha; Lancaster University, Spectrum Centre for Mental Health Research, Division of Health Research<br>Rycroft-Malone, Jo; Lancaster University, Faculty of Health and Medicine<br>Slade, Mike; University of Nottingham School of Health Sciences; Nord University, Faculty of Medicine and Health Sciences<br>Whittaker, Lesley; Lancashire and South Cumbria NHS Foundation Trust, Patient Experience, Engagement & Safeguarding<br>Lobban, Fiona; Lancaster University, Spectrum Centre for Mental Health Research, Division of Health Research
**Primary Subject Heading**:	Mental health
Secondary Subject Heading:	Health services research, Qualitative research
Keywords:	QUALITATIVE RESEARCH, Social Support, MENTAL HEALTH

SCHOLARONE™
Manuscripts

I, the Submitting Author has the right to grant and does grant on behalf of all authors of the Work (as defined in the below author licence), an exclusive licence and/or a non-exclusive licence for contributions from authors who are: i) UK Crown employees; ii) where BMJ has agreed a CC-BY licence shall apply, and/or iii) in accordance with the terms applicable for US Federal Government officers or employees acting as part of their official duties; on a worldwide, perpetual, irrevocable, royalty-free basis to BMJ Publishing Group Ltd ("BMJ") its licensees and where the relevant Journal is co-owned by BMJ to the co-owners of the Journal, to publish the Work in this journal and any other BMJ products and to exploit all rights, as set out in our licence.

The Submitting Author accepts and understands that any supply made under these terms is made by BMJ to the Submitting Author unless you are acting as an employee on behalf of your employer or a postgraduate student of an affiliated institution which is paying any applicable article publishing charge ("APC") for Open Access articles. Where the Submitting Author wishes to make the Work available on an Open Access basis (and intends to pay the relevant APC), the terms of reuse of such Open Access shall be governed by a Creative Commons licence – details of these licences and which Creative Commons licence will apply to this Work are set out in our licence referred to above.

Other than as permitted in any relevant BMJ Author's Self Archiving Policies, I confirm this Work has not been accepted for publication elsewhere, is not being considered for publication elsewhere and does not duplicate material already published. I confirm all authors consent to publication of this Work and authorise the granting of this licence.

For peer review only

TITLE: Designing a Library of Lived Experience for Mental Health: integrated realist synthesis and experience-based co-design study in UK mental health services

Word Count: 4308

Authors and affiliations

Corresponding author: Paul Marshall, Spectrum Centre for Mental Health Research, Lancaster University, Lancaster, UK, p.marshall4@lancaster.ac.uk

John Barbrook, Lancaster University Library, Lancaster, UK

Grace Collins, freelance participatory artist, UK

Sheena Foster, Spectrum Centre for Mental Health Research, Lancaster University, Lancaster, UK

Zoe Glossop, Spectrum Centre for Mental Health Research, Lancaster University, Lancaster, UK

Clare Inkster, NHS England North West, Manchester, UK

Paul Jebb, Patient Experience, Engagement & Safeguarding, Lancashire and South Cumbria NHS Foundation Trust, Preston

Rose Johnston, Spectrum Centre for Mental Health Research, Lancaster University, Lancaster, UK

Steven Jones, Spectrum Centre for Mental Health Research, Lancaster University, Lancaster, UK

Hameed Khan, Spectrum Centre for Mental Health Research, Lancaster University, Lancaster, UK

Christopher Lodge, Spectrum Centre for Mental Health Research, Lancaster University, Lancaster, UK

Karen Machin, independent survivor researcher, UK

Erin E. Michalak, Department of Psychiatry, University of British Columbia, Vancouver, British Columbia, Canada

Sarah Powell, Lancaster Medical School, Lancaster University, Lancaster, UK

Samantha Russell, Spectrum Centre for Mental Health Research, Lancaster University, Lancaster, UK

Jo Rycroft-Malone, Faculty of Health and Medicine, Lancaster University, Lancaster, UK

Mike Slade, School of Health Sciences, Institute of Mental Health, University of Nottingham, Nottingham, UK; Faculty of Medicine and Health Sciences, Nord University, Namsos, Norway

Lesley Whittaker, Patient Experience, Engagement & Safeguarding, Lancashire and South Cumbria NHS Foundation Trust, Preston, UK

Fiona Lobban, Spectrum Centre for Mental Health Research, Lancaster University, Lancaster, UK

For peer review only

For peer review only - http://bmjopen.bmj.com/site/about/guidelines.xhtml

ABSTRACT

Objective

Living Library events involve people being trained as living 'Books', who then discuss aspects of their personal experiences in direct conversation with attendees, referred to as 'Readers'. This study sought to generate a realist programme theory and a theory-informed implementation guide for a Library of Lived Experience for Mental Health (LoLEM).

Design

Integrated realist synthesis and experience-based co-design.

Setting

Ten online workshops with participants based in the North of England.

Participants

Thirty-one participants with a combination of personal experience of using mental health services, caring for someone with mental health difficulties, and/or working in mental health support roles.

Results

Database searches identified 30 published and grey literature evidence sources which were integrated with data from 10 online co-design workshops conducted over 12 months. The analysis generated a programme theory comprising five context-mechanism-outcome (CMO) configurations. Findings highlight how establishing psychological safety is foundational to productive Living Library events (CMO1). For Readers, direct conversations humanise others' experiences (CMO 2) and provide the opportunity to flexibly explore new ways of living (CMO 3). Through participation in a Living Library, Books may experience personal empowerment (CMO 4), while the process of self-authoring and co-editing their story (CMO 5) can contribute to personal development. This programme theory informed the co-design of an implementation guide highlighting the importance of tailoring event design and participant support to the contexts in which LoLEM events are held.

Conclusions

The LoLEM has appeal across stakeholder groups and can be applied flexibly in a range of mental health-related settings. Implementation and evaluation are required to better understand the positive and negative impacts on Books and Readers.

Registration

PROSPERO CRD42022312789

Strengths and limitations of this study

- This study used a novel, iterative, and creative approach to integrating theory development and intervention co-design.
- A key strength of this approach was the involvement of people with lived experience expertise in mental health at every stage of co-design and theory development.
- The programme theory and implementation guidance were informed by analysis of research on previous Living Libraries and detailed co-design workshops, which drew on broad professional and personal mental health experiences.

For peer review only - http://bmjopen.bmj.com/site/about/guidelines.xhtml

- However, few evidence sources identified by systematic searches describe Living Libraries focused specifically on mental health.

Keywords

Mental Health Services, Psychosocial Intervention, Qualitative Research, Social Support

For peer review only

For peer review only - http://bmjopen.bmj.com/site/about/guidelines.xhtml

INTRODUCTION

The value of sharing health related experiences is widely recognised. Varied contexts draw on these experiences, including for shaping research [1], enriching professional education [2], and informing peer support [3]. There is an expanding evidence-base focused specifically on mental health lived experiences. For example, social contact interventions reduce mental health stigma [4], mental health peer support contributes to improvements in psychosocial outcomes [5], and there is growing interest in the use of mental health narratives to achieve a range of organisational aims [6]. The importance of integrating lived experience perspectives in health service development is acknowledged in UK and global policy [7,8], emphasising the need for continued efforts to promote lived experience perspectives.

Existing models for sharing mental health experiences do have limitations. Many opportunities are offered only periodically by health services and educational institutions, such as those linked to service improvement [9] or health professional training [10]. Others require commitment to more formal positions, including the peer-support worker role in the UK [11], which may preclude access by those with existing commitments. There is an outstanding need to extend such opportunities to groups whose voices are often not heard in mainstream conversations about mental health, including those from marginalised communities [12].

Adaptable approaches are required for expanding opportunities to share and learn from mental health experiences. The Living Library may represent one such model. Pioneered by the Human Library Organisation [13], these events involve people being supported to share aspects of their life experience in direct conversations with others. Those sharing their stories are called living 'Books', while those listening are termed 'Readers'. Conversations often last around 20 minutes and involve a Book briefly describing their personal narrative, then being open to questions from the Reader [14]. Events are facilitated by staff, or 'Librarians', who provide guidance to those involved. These events are often held in open settings, including public and university libraries, and have typically aimed to challenge a range of societal prejudices by facilitating interactions between people who may not otherwise engage in conversation around topics such as personal experience of racial prejudice, experience of ill-health, and gender-based discrimination [15]. The model has also been used in a mental health context to address stigma [16] and facilitate peer-support [17]. However, implementation recommendations sensitive to the specific challenges of discussing mental health experiences are limited.

Following UK complex intervention development guidance calling for greater emphasis on theory development and stakeholder engagement [18], we applied a novel integration of realist synthesis and experience-based co-design (EBCD) to explore the Living Library as a strategy for sharing mental health experiences. Realist synthesis is a method of evidence synthesis used to develop causal statements, or programme theories, to explain how social programmes work, for whom and under which circumstances [19]. EBCD identifies opportunities for healthcare innovation and draws on stakeholder perspectives to generate creative solutions [20]. Our integrative approach is detailed in a published research protocol [21].

Objective

- To use realist synthesis to develop a programme theory for a mental health-focused Living Library, which we term a Library of Lived Experience for Mental Health (LoLEM).

For peer review only - http://bmjopen.bmj.com/site/about/guidelines.xhtml

To use insights from this synthesis to inform co-design workshops with a range of mental health stakeholders, with the goal of developing an accessible implementation guide.

METHODS

This study received ethical approval from Coventry and Warwick National Health Service Research Ethics Committee (ref: 305975). The systematic search strategy was developed with an information specialist (JB) and pre-registered on PROSPERO https://www.crd.york.ac.uk/prospero/display_record.php?RecordID=312789. RAMESES guidance informed the reporting of this study [22]. A COREQ checklist is presented in supplemental file 1.

As described in the study protocol [21], theory development and EBCD workstreams were iterative and ran in parallel. However, the study broadly progressed through the following stages which represent an integration of the steps taken when conducting realist synthesis and EBCD [18, 19] :

Eliciting initial theories and touchpoints

Initial programme theories (IPTs) were elicited through theory gleaning interviews [23] with 6 members of an expert advisory group and the study public and patient involvement (PPI) lead who were invited to contribute to the study based on their experience participating in or researching Living Libraries in the domains of mental health (EM, MS, SF, CL), education (SF) and stigma reduction (CI, HK) (topic guide available in supplemental file 2). Interviews were conducted and recorded using the online video software Microsoft Teams by PM, a male research associate with doctoral level experience in qualitative interviewing. PM had no previous experience of Living Libraries prior to conducting the interviews. As members of the research team, all interviewees had prior relationships with the interviewer and were fully informed of the reason for their participation. Twenty IPTs were developed based on initial coding by PM, FL and RJ. Research bias was mitigated through written feedback on initial drafts and advisory group discussion prior to the IPTs being finalised (supplemental file 3). IPTs highlighted key issues to be explored with participants in co-design workshops, referred to as 'touchpoints' in the EBCD process [20].

Retrieving relevant evidence

Searches were conducted on research databases and grey literature sources (figure 1) from 2000, the inception of the initial Human Library approach [13], to March 2023. Preparatory unsystematic searches indicated that relatively few articles in this literature were likely to focus solely on mental health. Therefore, reports on Living Libraries related to specific topics including mental health and from generalist events featuring a range of lived experiences were included. Following title and abstract screening, the full-texts of identified articles were judged for inclusion using a realist-informed assessment of relevance and rigour (PM) with 20% independently assessed by a second reviewer (RJ). Citation chaining was used to identify articles in reference sections and 'cited by' pages of articles included in the initial search. IPTs and initial workshops highlighted psychological safety as a key theoretical and implementation focus for this study. Consistent with the iterative nature of realist synthesis searches [24], we conducted a targeted search of a large multidisciplinary database for existing formal theories related to the concept and included an additional evidence source [25]. A full description of each search is available in supplemental file 4.

Iterating initial theories

For peer review only - http://bmjopen.bmj.com/site/about/guidelines.xhtml

Following guidance on the use of qualitative analysis software for realist analysis [26] , IPTs were added to NVivo 12 as nodes to create an initial programme theory framework. PM conducted coding of the full text of each evidence source. This involved identifying and labelling sections of text related to potential contextual factors, mechanisms, and/or outcomes, as defined in realist methodology [19]. Codes were linked to the most relevant initial programme theory node. PM, FL, and RJ refined the initial theory framework by reformulating, consolidating, and combining initial statements based on whether underlying data supported, refuted, or refined individual IPTs. While reviewing this evidence, retroductive reasoning was used to hypothesise causal forces behind regularities apparent in the data. An iterative process of written feedback and group discussion led to the consolidation of the initial framework into a series of candidate CMO configurations.

Establishing a multi-stakeholder co-design group

Eligible EBCD participants were adults with any self-identified mental health experience. We recruited participants by distributing digital study advertisements to local mental health services, health research networks, and third sector mental health support organisations primarily based in the North of England. Workshops were facilitated by KM and GC, both experts in mental health peer-support who deliver workshops from a lived experience perspective. Workshops were supported by researchers (ZG, RJ, PM), senior nurses (PJ, LW), a service user researcher (CL), and academic clinical psychologist (SJ).

Designing workshops reflexively

We ran 10, 2-hour online co-design workshops over 12 months using the online video software Zoom. Workshops were flexibly designed in team meetings around two primary goals. First, we aimed to develop a comprehensive implementation guide. This required the team to reflect on gaps in knowledge and plan further exploration of topics pertinent to implementation. Second, we explored 'touchpoints' to facilitate programme theory refinement. One such example was the exploration of psychological safety in workshop 4, a key concept in the IPT framework which required further investigation to understand relevant implementation factors.

Developing and refining outputs

Workshop participants' views and implementation suggestions were captured using the collaborative online note taking platform Jamboard [27] and in researcher (CL, PM, RJ, SJ, ZG) field notes. Data were combined in workshop summary documents following each session, reviewed by the research team in post-workshop de-briefs, and added to NVivo for integration into the ongoing analysis.

Summary documents informed an initial draft implementation guide, refined through feedback from the EBCD group. Implementation recommendations from the co-design process were linked to the developing theoretical framework. CMOs were further refined through discussion and written feedback from the wider research team and expert advisory group.

Final versions of the LoLEM programme theory and implementation guide were shared during an interactive dissemination event at which the LoLEM was piloted and the guide made freely available [28].

Public and patient involvement

Experts by experience in mental health contributed to all stages of this study. A stakeholder group informed the initial study design and experts by experience were co-applicants for funding. The

For peer review only - http://bmjopen.bmj.com/site/about/guidelines.xhtml

study team included a service user research and experts by experience were involved in an advisory group, all of whom are involved in authoring key study outputs. The co-design group involved service users and carers and was led by facilitators who delivered workshops from a lived experience perspective. Participants and team members with lived experience assisted with dissemination at local and national events.

RESULTS

Results of systematic searches

Database and grey literature searches returned 30 eligible evidence sources. Figure 1 describes the process of evidence identification. Study characteristics are available in supplemental file 5.

Figure 1. Flow diagram of evidence identification

[figure 1 here]

Description of co-design workshops

EBCD workshops were attended by 31 participants, 16 of whom identified as female and 15 male. Four had previously been involved in hosting a Living Library event and 1 had experienced being a living Book. Workshops were attended by people with experience of using mental health services (n=23), informal caregiving (n=13), and working in mental health services (n=8) or in voluntary sector mental health support roles (n=8). Participant demographic details are available in supplemental file 6.

Details of activities completed, and mental health experiences represented at each workshop, are presented in Table 1.

Table 1. Experience-based co-design workshop details

Workshop number	Total Participants	Experience using mental health services	Experience as informal carers	Experience as mental health service staff	Experience in voluntary mental health support roles	Workshop Activities
1	25	19	8	4	7	• Project introduction<br>• Story formulation activity
2	27	18	11	5	7	• Story sharing activity<br>• Reflection on personal boundaries
3	25	21	10	4	8	• Creating a fictional Reader 'persona'<br>• Reflection on Library design for different Reader groups
4	24	20	10	2	8	• What influences potential Readers' decisions to participate?<br>• What constitutes a safe space for sharing mental health experiences?<br>• How can event organisers cultivate psychological safety amongst Books and Readers?
5	25	19	11	2	8	• Reflection on participants' preferred roles at a Living Library (Books, Readers, or Librarians)

For peer review only - http://bmjopen.bmj.com/site/about/guidelines.xhtml

						• Planning a hypothetical Living Library<br>• A 'design to fail' activity, considering factors likely to contribute to unsuccessful events
6	23	17	8	3	6	• Design considerations for Living Libraries in different contexts (the voluntary, the NHS, and public libraries)
7	23	18	9	2	7	• Identifying resources required to run successful Living Libraries<br>• A 'sales pitch' activity to potential funders
8	21	16	9	2	5	• Advantages and disadvantages of delivering Living Libraries with different aims (mental health support versus changing public perception of mental health)<br>• Co-design of the implementation guide contents page
9	20	15	10	2	5	• The desired characteristics of event staff ('Librarians')<br>• Considering how to evaluate a Living Library<br>• Feedback on the implementation guide
10	17	15	7	2	3	• Celebration event<br>• Review of project successes and thanking participants for their contributions

LoLEM programme theory

We developed a programme theory comprising five CMO configurations, with corresponding implementation recommendations (Figure 2).

Figure 2. LoLEM programme theory with implementation recommendations

[figure 2 here]

CMO 1 – Psychological safety facilitates dialogue

When event organisers implement guidance and support strategies tailored for their organisational settings (context), participants will be better able to understand the Living Library model and how to manage personal boundaries such that they feel in control of their experience (mechanism). This will promote participants' perceptions of psychological safety (outcome) necessary for engaging and productive conversations.

Psychological safety refers to an individual's comfort with taking interpersonal risks to meet their goals within a given organisational context [25]. The Living Library model provides a platform for participants to take interpersonal risks by sharing experiences in direct conversation. We propose that psychological safety creates a facilitating context, or 'ripple' effect [29], promoting the likelihood of subsequent positive impacts described by CMOs 2-5.

Developing psychological safety for Books and Readers commonly involves implementing event rules to explicitly promote mutual respect, awareness of boundaries, and a sense of personal control. Pre-event briefings may be used to reinforce the principles of the approach: "'Readers are required to return the 'Book' in the same psychological and physical condition and are asked: 'Never harm a Book!'" [30]. Promoting psychological safety for Books can involve offering training and practice sessions to set expectations, identify what they wish to share, and prepare for questions they may not want to answer. EBCD participants suggested that psychological safety would further be

For peer review only

For peer review only - http://bmjopen.bmj.com/site/about/guidelines.xhtml

promoted by the reassuring presence of attentive staff, or 'Librarians', able to provide guidance, oversight, and emotional support where necessary.

Given their conversational focus, Living Libraries hold potential for Books and Readers to experience interpersonal challenges that could undermine psychological safety. Books may be exposed to negative attitudes towards mental health, feel pressure to be an exhaustive authority on the issues they discuss, 'pumped' for knowledge by repeated readings, or alternatively, 'left on the shelf' by uninterested Readers [31,32]. Readers themselves may experience discomfort hearing directly about others' distress. The emergent nature of these interactions means that such circumstances cannot be entirely predicted, yet event planners should prepare for and mitigate these eventualities as far as possible by foregrounding implementation strategies to promote psychological safety.

CMO 2 – Humanising others' experiences

Direct conversations with Books (context) humanise the experience of mental health difficulties (mechanism), contributing to Readers developing a greater empathetic understanding of mental health (outcome).

Event organisers framed direct interaction between Books and Readers as an explicit goal. Many aimed "to bring people together who may not otherwise meet and to also kind of shut down the stereotypes that people have about others" [33]. Events focused on challenging prejudice sought to provide a platform for Books to "show the general public they are 'human'" [34] and challenge superficial views of important aspects of their experiences and identities by breaking down perceived "us-them" divides between Books and Readers [35]. This could occur through recognition of shared experience including emotional difficulties, to which Readers could relate regardless of personal differences: "Even women [who don't] look like me, or identify as the same race as me still go through similar struggles. So that was eye-opening because I've never really thought about it like that" [35].

This more nuanced understanding of others' experiences was for some facilitated by the subtle emotional and behavioural cues present in direct interaction, which augmented the interpersonal connection shared by Books and Readers [33,36]. In this context, the experiences and identities Books represent shift from abstract and disembodied concepts to personified and meaningful human experiences [37,38]. Mental health difficulties are thus re-conceptualised from "myths to storied realities" [39],, enhancing Readers' abilities to subsequently understand and empathise with the perspectives of people experiencing similar difficulties [35].

CMO 3 – Exploring new ways of living

When Living Libraries provide Readers with the permission to explore Books' experiences through synchronous interaction (context), they will use this opportunity to flexibly explore issues of personal significance (mechanism). This will facilitate awareness of new and helpful ways of living (outcomes).

The synchronous nature of Living Library conversations allows Readers to personalise their interactions by asking questions about aspects of Books' stories that resonate with their own. As noted by a participant in a mental health-specific event [35], "in a talk, listeners listen passively. As speakers, we are asked to tell our whole story. But in the Human Library, it does not matter whether the sharing is complete or not. The important thing is to let readers know what they want to know."

For peer review only - http://bmjopen.bmj.com/site/about/guidelines.xhtml

This form of experiential learning can influence how Readers understand ways of managing their own distress [17] and may help health professionals identify new ways of supporting service users [37,40].

EBCD participants noted that the relative novelty of the Living Library approach and the associated ‘Book’ metaphor can imply that the sharing of experience is intended to be unidirectional, with Readers taking a passive role. Organisers can facilitate interactive dialogue by providing Readers with explicit permission to explore questions of personal significance, within established boundaries. Strategies for reaffirming Readers’ conversational permission include providing library attendees with clear ground rules and example questions or cues to prompt engagement.

CMO 4 – Personal empowerment

When Living Libraries facilitate the authentic expression of Books’ personal experiences (context), Books will feel that their expertise has been heard and valued (mechanism), contributing to personal empowerment (outcome).

Living Libraries have been used to spotlight marginalised voices [41]. The approach both promotes Books’ sense of having been heard as experts by experience and provides a medium to use their stories to meet personally valued goals, including shaping public attitudes and offering support to others. Personal empowerment therefore emerges from this opportunity for direct self-representation, recognition, and the pursuit of positive change [31,42]. EBCD group members highlighted the potential of a LoLEM to meet the motivation of many experts by experience in mental health to use their unique perspectives to inspire individual, organisational, and social progress against a wider context of stigma and under-recognition. This was mirrored in literature describing participants’ sense of pride and satisfaction after sharing their stories at Living Library events [38,39,43].

The EBCD group also identified how personal empowerment can occur through meaningful participation in event design and delivery. Extensive involvement of experts by experience was suggested to diminish power imbalances between those with lived experience and organisations, such as health services and universities, that may host a LoLEM. Workshops highlighted that the greater the degree of lived experience involvement and collaborative working alongside staff, the more likely a LoLEM is to reflect the perspective of the groups it seeks to engage. It was suggested that this may reduce the potential for disempowerment emerging from peoples’ stories being misused.

CMO 5 – Self authoring and co-editing

When Living Libraries support Books with developing a personal narrative to share in conversation with Readers (context), Books will explore and develop insight into these aspects of their lives (mechanism). This leads Books to develop new ways of understanding and sharing their experiences which can contribute to personal growth (outcome).

For Books, authoring and articulating a personal narrative represents a “self-directed process of discovery” [31] and ongoing engagement with story sharing can “demonstrate how their personal identities evolve and develop over time” [43]. This process may contribute to the reframing of mental health difficulties as an aspect of past experience that is of value to the present self [39].

For peer review only - http://bmjopen.bmj.com/site/about/guidelines.xhtml

For peer review only

Relatively unstructured dialogue may also facilitate a form of narrative co-editing: "…in the process of creating a narrative in cooperation with readers, books actually alter their understanding of their own self-appointed topic and what it means to them" [31]. Personal growth can therefore result from a shift in the Books' perceptions of their current circumstances through reflexive story refinement: "Despite occasional moments of discomfort and, perhaps in some cases, because of them, Books recognized that their stories changed because of their participation in the HL [Human Library] Project. Details were added, elements they believed were less important emerged as such, and overall, they achieved greater clarity about their narratives" [34].

Table 2 provides illustrative data supporting each CMO.

Table 2 Programme theories with illustrative data extracts

For peer review only

Programme theory	Illustrative data	Source
1. Psychological safety facilitates dialogue	"[Books'] needs must be recognised and met. This could include things like limiting the number of readings a living book can be required to participate in within a given session, providing adequate support for living books including those with special needs, and providing time and structure for living books to debrief after readings or to read other living books"	[31]
	" [a living library conversation] will be a deep dive, might need to help these first-time Books prepare e.g. with practice and storytelling and thinking about boundaries"	Workshop 2 Jamboard
2. Humanising others' experiences	"Readers were given permission to empathize with the experiences of Books and, in the process, see themselves in the Books' stories"	[34]
	"Stories trigger a response in us that helps us empathise with others"	Workshop 3 field notes
3. Exploring new ways of living	"Another Reader found inspiration in a Book's journey that he could apply to his own life: "I learned from some people's experiences that, okay, I'm going through this right now, but you went through it and you overcame it. So I have a choice."	[42]
	"[Books are] providing other visions/real life examples of ways forward that are hopeful"	Workshop 8 Jamboard
4. Personal empowerment	"Living libraries excel as a strategy for giving voice to marginalised groups. Living library conversations allow for direct self-representation, unmediated by third parties."	[41]
	"Mental health has been stigmatised for so long, people ignored, locked away and forgotten. Just the very action of telling someone that you want to hear their story is massive."	Workshop 2 Jamboard
5. Self-authoring and co-editing	"By thinking about and structuring their story, people will often 're-author' their lives, by defining their own existence in relationship to themselves and what they were going through at	[44]

For peer review only - http://bmjopen.bmj.com/site/about/guidelines.xhtml

	the time; thereby constructing reality by choices made to give meaning to their lives”	
	“Storytelling can be therapeutic for teller”	Workshop 2 Jamboard

Implementation guidance

The integrated realist synthesis and EBCD process contributed to the development of a theory informed implementation guide. Recommendations are summarised here with reference to key stages of event delivery (Table 3). The full guide is available online [28].

Table 3. Co-designed implementation recommendations by event delivery stages

	Set-up
Team building (CMO1, CMO4)	Ensure the event team includes people with the skills to plan, promote, and implement a mental health-related event. Embed the perspectives of experts by experience, including in the mechanism by which stories are selected. Recruit books with diverse relevant experiences. Where applicable, provide context-appropriate and timely payment.
Goal setting (CMO1)	Identify a clear goal for the event. Align this with plans for recruitment, support provision, and promotion.
Venue selection (CMO1)	Select a non-stigmatising venue that provides a degree of privacy for individual discussions yet does not leave participants feeling isolated and exposed in conversations with people they may not have met before. Conduct accessibility and safeguarding assessments.
	Pre-event
Book training (CMO5)	Provide comprehensive training to Books. Focus on helping Books to understand the model, what is expected of them, and where to go for support. Clearly identify personal boundaries for story sharing and how to manage them. Generate a synopses and story title. Consider developing individualised support plans.
Team practice (CMO1, CMO5)	Rehearse how the Living Library will be delivered. Books may wish to practice sharing their stories with team members and/or each other to refine their story and prepare for the event.
Develop materials (CMO4)	Develop event materials alongside experts by experience. This may include guidance for readers highlighting what the event is for, rules for participation, and support materials.
	At the Library
Ground-rules (CMO1, CMO2, CMO3)	Clearly outline ground-rules for conversations. This may include instructions for engaging in respectful dialogue and for Books and Readers to be mindful of any boundaries for their conversations, such as to avoid certain topics. Encourage readers to explore topics and questions of personal interest within these boundaries.

For peer review only - http://bmjopen.bmj.com/site/about/guidelines.xhtml

Content Warnings (CMO1)	If appropriate, consider generating content warnings which could be included in Books' synopses.
Attentive support (CMO1)	Ensure that emotional support is consistent with the nature and scale of the event. Develop plans for managing distress, including risk-related scenarios.
	Post-event
De-briefing (CMO1)	Implement individual or group debriefs. Consider providing Readers with the opportunity to reflect on their experiences, for example in a quite space away from the main event.
Signposting (CMO1)	If appropriate, make available relevant and up-to-date sign-posting material.
Supervision (CMO1, CMO5)	Consider ongoing supervision for Books and Librarians. This could include a review of any difficulties, support needs, or changes to Books' stories. Where available, discuss further opportunities for lived-experience involvement within the organisation.

DISCUSSION

This integrated realist synthesis and EBCD study generated a programme theory for a LoLEM and theory-informed implementation recommendations. Results emphasise the importance of psychological safety for facilitating productive conversations between Books and Readers. Implementation recommendations highlight ways in which potential organisers may seek to foster supported environments for sharing mental health experiences and further suggestions are reported in a co-designed implementation guide [28].

This study builds on implementation recommendations for the Human Library Organisation's generalist approach [14]. A notable difference in the context of mental health is the centrality of psychological safety, defined as the ability to take desired interpersonal risks. Occupational research indicates that psychological safety is associated with proactive communication including concern-raising within teams, individual and team-level learning, and work engagement [25]. Recommendations for promoting psychological safety within this literature suggest organisations should seek to create cultures defined by collaboration and interpersonal openness [45]. This aligns with established principles-based approaches to mental health peer-support [46], which point to the significance of safe and trusting relationships within organisations that draw on lived experience, and control over how those experiences are shared. Results here suggest that by investing in training and building meaningful relationships with those sharing their stories, LoLEM organisers can clearly articulate expectations and promote informed personal disclosure. Drawing on established measures [25], further research could evaluate the extent to which these practices influence Books' perceptions of psychological safety.

Values underpinning mental health peer support, such as mutuality and reciprocity [47], are reflected in evidence of positive impacts amongst those delivering and accessing these services. For example, benefits for those receiving support include improvements in social functioning, hope, and stigma reduction, while peer supporters may experience greater confidence, skills development, and positive reframing of personal identity [48]. Building on this evidence, results of this study suggest that a LoLEM could provide opportunities to learn about ways of understanding and managing mental health from people who may be empowered by being supported to share their stories.

For peer review only - http://bmjopen.bmj.com/site/about/guidelines.xhtml

Indeed, participants in a Living Library focused on bipolar self-management welcomed the opportunity for direct contact with peers [17], demonstrating the feasibility of using this approach in the peer support context. A LoLEM could also assist service users with sharing stories as part of a recovery-oriented programme of support [35]. Indeed, there is an established body of literature describing the use of storytelling in mental health [6,49] indicating that developing a personal narrative can be highly meaningful for the teller, yet also potentially emotionally challenging [50]. This reinforces this study's emphasis on dedicated support with story formulation for potential Books.

The LoLEM approach requires adaptation for specific contexts. Organisers should consider factors that could undermine the safe and empowering implementation of lived-experience focused programmes. Survivor research identifies how power imbalance between those with lived experience and health services could serve to discourage perspectives deemed unacceptable to institutional agendas, including critical and marginalised voices, or delegitimize those who draw on their experience in pursuit of systemic change [51]. A recent systematic review of lived experience narratives drew attention to potential misuses of mental health experiences, including commodification, coerced elicitation, harm to narrators or audiences, and curatorial decisions that limit diversity [6]. Good practice recommendations relevant to the LoLEM model include proactive reflection on organisers' personal biases to ensure power imbalances are not reinforced, encouraging audiences to endorse ethical listening and openness to difference, seeking consent, and promoting control over personal narratives [6]. Consistent with this, findings here emphasise the vital role of lived experience perspectives in implementing transparent and collaborative LoLEMs. Established models for involvement, including EBCD, could be used to facilitate development of future programmes.

Implications for research

Further work may explore the feasibility of Living Libraries as sustained programmes and whether effects persist over time. CMOs reported here could inform the selection of outcomes. The humanising impact of a LoLEM would be expected to contribute to more empathetic perspectives on mental health and in the context of peer-support, future research may investigate potential impacts on Reader self-efficacy. For Books, the proposed positive impact of storytelling and personal empowerment aligns with definitions of personal recovery for which several assessments have been developed [52]. Factors underpinning differences in outcomes across varied service settings, and how this model may fit alongside existing lived experienced-based interventions, are currently unknown. These issues could be addressed using realist evaluation methodology.

Implications for practice

While lived experience opportunities continue to grow within health services, most roles do not involve training in self-disclosure [53]. The LoLEM model could bridge the gap between people with mental health experiences seeking opportunities to help others, and more formal paid positions requiring considerable time commitments. The approach could also complement efforts to increase lived experience expertise in health professional training, as evidenced by its successful implementation in social work training [37]. More specifically, a LoLEM could afford healthcare trainees opportunities to engage with a broader range of people than they may typically meet, and in neutral settings outside of clinical environments which may be more conducive to open conversation. It is also feasible to combine a Living Library with structured reflexive activities to embed learning and consider the applicability of a Book's story to a Reader's clinical practice [40]. Actions to promote equality, inclusion and diversity in the UK health service should apply an

For peer review only - http://bmjopen.bmj.com/site/about/guidelines.xhtml

intersectional lens [54]. It has been noted that the Living Library model is well suited to assist health professional trainees with developing a lived appreciation of intersectionality through conversations that illuminate how aspects of identity interact [34]. As per the programme theory reported here a LoLEM could serve to humanise and promote the understanding of nuanced relationships between mental health experiences and other salient aspects of identity.

Strengths and limitations

A strength of this approach is the breadth of mental health experience represented in the study team. PPI perspectives were sought throughout, including during study design, via an advisory group and in co-design meetings, and to assist with dissemination. This facilitated the generation of findings that reflect a variety of perspectives and contexts in which a LoLEM could be hosted. This project also demonstrates the feasibility of a novel and creative approach to mental health programme co-design and theory generation. However, this study does have limitations. Many evidence sources identified in systematic searches were non-mental health specific and involved short-term evaluations. This study principally recruited from the North of England which may limit the transferability of its findings. In a change to the study protocol [21], theory refinement interviews [23] were not conducted due to limitations in available resources, potentially limiting the explanatory depth of the final programme theory. The reported search on psychological safety was similarly limited to a single multidisciplinary database due to a limited capacity to manage the screening burden of a larger search.

CONCLUSION

This project is the first to apply realist methods to articulate explanations for how Living Libraries may work and adds to existing literature by identifying specific strategies for promoting the safety and effectiveness of this model as applied to mental health experiences. Continued recognition of the importance of lived experience in shaping mental health policy and practice justifies further consideration of how this approach can be implemented and evaluated.

FUNDING

This work was funded by the National Institute for Health and Care Research (NIHR) under its Research for Patient Benefit (RfPB) Programme (Grant Reference Number NIHR203476). The views expressed are those of the author(s) and not necessarily those of the NIHR or the Department of Health and Social Care. The study was sponsored by Lancashire and South Cumbria NHS Foundation Trust.

COMPETING INTEREST STATEMENT

None declared.

ETHICS APPROVAL

This study involved human participants and was granted ethical approval by Coventry and Warwick National Health Service Research Ethics Committee on 29th December 2021 (REF: 305975). Participants gave informed consent to participate in the study before taking part.

DATA AVAILABILITY STATEMENT

No data are available.

For peer review only - http://bmjopen.bmj.com/site/about/guidelines.xhtml

AUTHOR CONTRIBUTIONS

FL designed and led the study. PM conducted data collection and analysis for the realist synthesis. JB supported search strategy development. KM and GC co-facilitated EBCD workshops with support from SJ, RJ, ZG, CL, PJ and LW. JR-M provided methodological guidance for the realist synthesis. SF, CI, HK, SP, EM, and MS formed an expert advisory group which provided strategic guidance across the study. SR supported article writing. All authors approved the final article.

ORCID IDs

Mike Slade 0000000170203434

Steven Jones 0000-0002-8801-5113

Samantha J Russell 0000-0003-1904-9739

Paul Marshall 0000-0002-3913-3359

Sarah Powell 0000-0002-3509-5789

Zoe Glossop 0000-0002-6151-4860

Fiona Lobban 0000-0001-6594-4350

TWITTER

Steven Jones @lancsspectrum

Sarah Powell @DrSarahPowell1

John Barbrook @stackingbooks

Paul Jebb @pauljebb1

Lesley Whittaker @Lesleyp222

ACKNOWLEDGMENTS

The research team would like to thank all those who contributed to the co-design of the outputs for this study.

REFERENCES

1 Capobianco L, Faija C, Cooper B, *et al.* A framework for implementing Patient and Public Involvement in mental health research: The PATHWAY research programme benchmarked against NIHR standards. *Health Expect*. 2023.

2 Rowland P, Anderson M, Kumagai AK, *et al.* Patient involvement in health professionals' education: A meta-narrative review. *Adv Health Sci Educ*. 2019;24:595–617.

3 Shalaby RAH, Agyapong VI. Peer support in mental health: literature review. *JMIR Ment Health*. 2020;7:e15572.

For peer review only - http://bmjopen.bmj.com/site/about/guidelines.xhtml

4 Maunder RD, White FA. Intergroup contact and mental health stigma: A comparative effectiveness meta-analysis. *Clin Psychol Rev*. 2019;72:101749.

5 White S, Foster R, Marks J, *et al.* The effectiveness of one-to-one peer support in mental health services: a systematic review and meta-analysis. *BMC Psychiatry*. 2020;20:1–20.

6 Yeo C, Rennick-Egglestone S, Armstrong V, *et al.* Uses and misuses of recorded mental health lived experience narratives in healthcare and community settings: systematic review. *Schizophr Bull*. 2022;48:134–44.

7 National Health Service. The NHS Long Term Plan. 2019. https://www.longtermplan.nhs.uk/wp-content/uploads/2019/08/nhs-long-term-plan-version-1.2.pdf

8 World Health Organization. Comprehensive mental health action plan 2013–2030. 2021.

9 Veldmeijer L, Terlouw G, Van Os J, *et al.* The Involvement of Service Users and People With Lived Experience in Mental Health Care Innovation Through Design: Systematic Review. *JMIR Ment Health*. 2023;10:e46590.

10 Happell B, Byrne L, McAllister M, *et al.* Consumer involvement in the tertiary-level education of mental health professionals: A systematic review. *Int J Ment Health Nurs*. 2014;23:3–16.

11 Beales A, Wilson J. Peer support–the what, why, who, how and now. *J Ment Health Train Educ Pract*. 2015;10:314–24.

12 Gillard S. Guest editorial: re-balancing the discourse, inviting critique and welcoming marginalised voices in research about peer support in mental health services. Adv. Ment. Health. 2022;20:87–90.

13 Human Library Organisation. About the Human Library. https://humanlibrary.org/about/

14 Little N, Nemutlu G, Magic J, *et al.* Don’t judge a book by its cover! *Living Libr Organiser’s Guide*. 2011.

15 Lam GYH, Wong HT, Zhang M. A Systematic Narrative Review of Implementation, Processes, and Outcomes of Human Library. *Int J Environ Res Public Health*. 2023;20. doi: 10.3390/ijerph20032485

16 Chung EY-H, Tse TT-O. Effect of human library intervention on mental health literacy: a multigroup pretest-posttest study. *BMC Psychiatry*. 2022;22:73.

17 Michalak EE, Morton E, Barnes SJ, *et al.* Supporting Self-Management in Bipolar Disorder: Mixed-Methods Knowledge Translation Study. *JMIR Ment Health*. 2019;6:e13493.

18 Skivington K, Matthews L, Simpson SA, *et al.* A new framework for developing and evaluating complex interventions: update of Medical Research Council guidance. *BMJ*. 2021;374.

19 Hunter R, Gorely T, Beattie M, *et al.* Realist review. *Int Rev Sport Exerc Psychol*. 2022;15:242–65.

20 Green T, Bonner A, Teleni L, *et al.* Use and reporting of experience-based codesign studies in the healthcare setting: a systematic review. *BMJ Qual Saf*. 2020;29:64–76.

For peer review only - http://bmjopen.bmj.com/site/about/guidelines.xhtml

21 Lobban F, Marshall P, Barbrook J, *et al.* Designing a library of lived experience for mental health (LoLEM): protocol for integrating a realist synthesis and experience based codesign approach. *BMJ Open*. 2023;13:e068548.

22 Wong G, Greenhalgh T, Westhorp G, *et al.* RAMESES publication standards: realist syntheses. *BMC Med*. 2013;11:1–14.

23 Mukumbang FC, Marchal B, Van Belle S, *et al.* Using the realist interview approach to maintain theoretical awareness in realist studies. *Qual Res*. 2020;20:485–515.

24 Booth A, Carroll C. Systematic searching for theory to inform systematic reviews: is it feasible? Is it desirable? *Health Inf Libr J*. 2015;32:220–35.

25 Newman A, Donohue R, Eva N. Psychological safety: A systematic review of the literature. *Hum Resour Manag Rev*. 2017;27:521–35.

26 Gilmore B, McAuliffe E, Power J, *et al.* Data analysis and synthesis within a realist evaluation: toward more transparent methodological approaches. *Int J Qual Methods*. 2019;18:1609406919859754.

27 Google. Jamboard. https://jamboard.google.com/

28 Marshall P, Jones S, Lodge C, *et al.* *Library of Lived Experience for Mental Health Implementation Guide*. 2023. https://doi.org/10.5281/zenodo.7920240

29 Jagosh J, Bush PL, Salsberg J, *et al.* A realist evaluation of community-based participatory research: partnership synergy, trust building and related ripple effects. *BMC Public Health*. 2015;15:1–11.

30 Bagci SC, Blazhenkova O. Unjudge Someone: Human Library as a Tool to Reduce Prejudice toward Stigmatized Group Members. *Basic Appl Soc Psychol*. 2020;42:413–31.

31 Ashmore A. Alive with knowledge: Engaging communities through living libraries. *Libr Stud J*. 2010;5:14–14.

32 Dreher T, Mowbray J. The power of one on one: Human libraries and the challenges of antiracism work. 2012.

33 Huang Y, Dobreski B, Xia H. Human library: Understanding experience sharing for community knowledge building. *Proceedings of the 2017 ACM conference on computer supported cooperative work and social computing*. 2017;1152–65.

34 Giesler MA. Humanizing Oppression: The Value of the Human Library Experience in Social Work Education. *J Soc Work Educ*. Published Online First: 2021.

35 Kwan CK. A Qualitative Inquiry into the Human Library Approach: Facilitating Social Inclusion and Promoting Recovery. *Int J Environ Res Public Health*. 2020;17.

36 Blizzard K, Becker Y, Goebel N. Bringing Women's Studies to Life: Integrating a Human Library into Augustana's Women's Studies Curriculum. *Coll Q*. 2018;21:n3.

37 Sen R, McClelland N, Jowett B. Belonging to the library: humanising the space for social work education. *Soc Work Educ*. 2016;35:892–904.

For peer review only - http://bmjopen.bmj.com/site/about/guidelines.xhtml

38 Sen R, Nylund M, Ali Hayward R, *et al.* The Living Library in social work education. *Involv Serv Users Soc Work Educ Res Policy Comp Eur Anal*. 2021;73.

39 Fortune D, Leighton J. Mental illness as a valued identity: How a leisure initiative promoting connection and understanding sets the stage for inclusion. *Leis Sci*. Published Online First: 25 March 2022. doi: 10.1080/01490400.2022.2058657

40 Inkster M C; St Jean, L; Elliott, P; Leach. Challenging Prejudice in Healthcare – the Human Library. 2016. https://www.nwpgmd.nhs.uk/sites/default/files/The%20Human%20Library%20for%20Healthcare%20Learners%20%282%29.pdf

41 Garbutt R. The living library: some theoretical approaches to a strategy for activating human rights and peace. *Act Hum Rights Peace Univers Responsib Conf 2008 Conf Proc Aust*. 2008;270–8.

42 Giesler M. Human Libraries. *New Soc Work*. 2020;27:24–8.

43 Watson GJ. “You shouldn’t have to suffer for being who you are”: An Examination of the Human Library Strategy for Challenging Prejudice and Increasing Respect for Difference. 2015.

44 Russell E, Linsley P. Human libraries: removing stigma. *Br J Wellbeing*. 2011;2:15–7.

45 Edmondson AC, Lei Z. Psychological safety: The history, renaissance, and future of an interpersonal construct. *Annu Rev Organ Psychol Organ Behav*. 2014;1:23–43.

46 Gillard S, Foster R, Gibson S, *et al.* Describing a principles-based approach to developing and evaluating peer worker roles as peer support moves into mainstream mental health services. *Ment Health Soc Incl*. 2017;21:133–43.

47 Repper J, Aldridge B, Gilfoyle S, *et al. Peer support workers: Theory and practice*. Centre for Mental Health London 2013.

48 Shalaby RAH, Agyapong VI. Peer support in mental health: literature review. *JMIR Ment Health*. 2020;7:e15572.

49 De Vecchi N, Kenny A, Dickson-Swift V, *et al.* How digital storytelling is used in mental health: A scoping review. *Int J Ment Health Nurs*. 2016;25:183–93.

50 Nurser KP, Rushworth I, Shakespeare T, *et al.* Personal storytelling in mental health recovery. *Ment Health Rev J*. 2018;23:25–36.

51 Kalocsai C, Agrawal S, de Bie L, *et al.* Power to the people? A co-produced critical review of service user involvement in mental health professions education. *Adv Health Sci Educ*. 2023;1–28.

52 Van Weeghel J, van Zelst C, Boertien D, *et al.* Conceptualizations, assessments, and implications of personal recovery in mental illness: A scoping review of systematic reviews and meta-analyses. *Psychiatr Rehabil J*. 2019;42:169.

53 Opie JE, McLean SA, Vuong AT, *et al.* Training of Lived Experience Workforces: A Rapid Review of Content and Outcomes. *Adm Policy Ment Health Ment Health Serv Res*. 2023;50:177–211.

For peer review only - http://bmjopen.bmj.com/site/about/guidelines.xhtml

54 NHS. NHS equality, diversity, and inclusion improvement plan. https://www.england.nhs.uk/long-read/nhs-equality-diversity-and-inclusion-improvement-plan/#interventions-by-protected-characteristic

For peer review only

For peer review only - http://bmjopen.bmj.com/site/about/guidelines.xhtml

TITLE: Designing a Library of Lived Experience for Mental Health: integrated realist synthesis and experience-based co-design study

Figure 1. Flow diagram of evidence deification selection

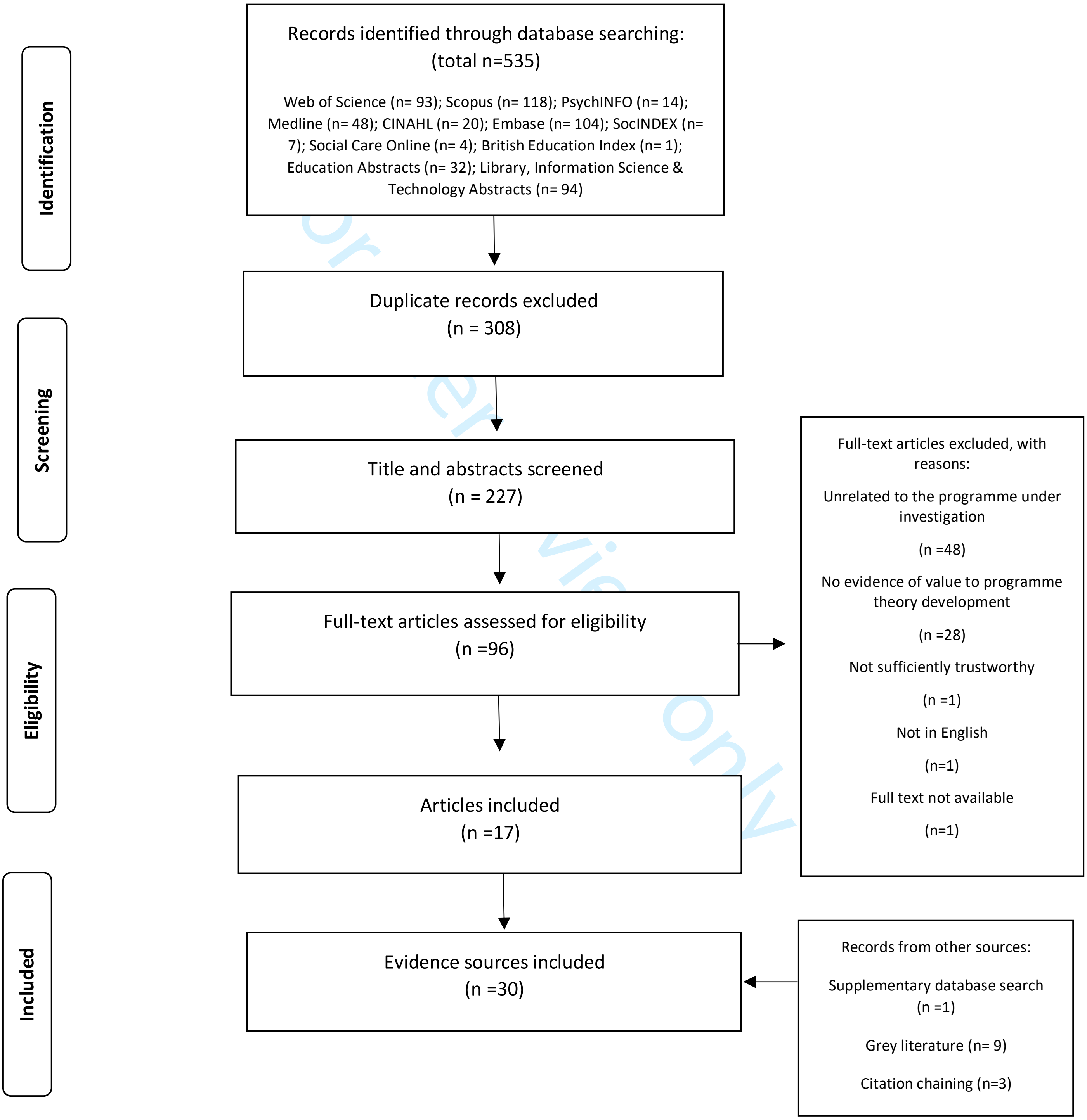

TITLE: Designing a Library of Lived Experience for Mental Health: integrated realist synthesis and experience-based co-design study

Figure 2. LoLEM programme theory with implementation recommendations

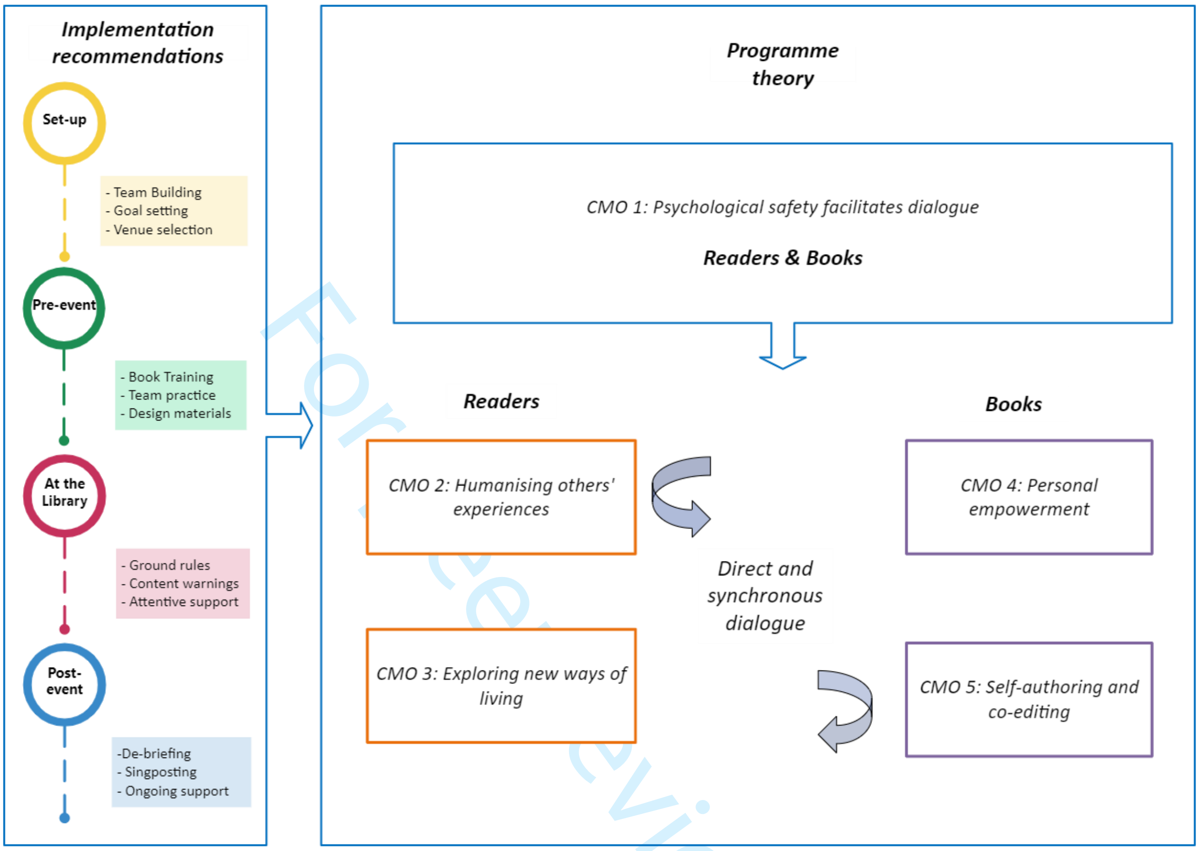

TITLE: Designing a Library of Lived Experience for Mental Health: integrated realist synthesis and experience-based co-design study

Supplemental file 1 – COREQ checklist

No. Item	Guide questions/description	Reported on Page #
**Domain 1: Research team and reflexivity**		
Personal Characteristics		
1. Inter viewer/facilitator	Which author/s conducted the interview or focus group?	Interviews were conducted by the lead author (PM) (page 5 line 19)
2. Credentials	What were the researcher's credentials? E.g. PhD, MD	PhD (corresponding author information)
3. Occupation	What was their occupation at the time of the study?	Research Associate (page 5 line 19)
4. Gender	Was the researcher male or female?	Male (page 5 line 22)
5. Experience and training	What experience or training did the researcher have?	Doctoral training in qualitative methods (page 5 line 20)
Relationship with participants		
6. Relationship established	Was a relationship established prior to study commencement?	Interviewees were members of a study advisory group and co-authors and as such, there was an established relationship with the researcher conducting the interview (page 5 line 21-22).
7. Participant knowledge of the interviewer	What did the participants know about the researcher? e.g. personal goals, reasons for doing the research	Interviewees were fully informed of the nature of the study prior to participation, including the researcher's aims for the interview and research study more broadly (page 5 line 21-22)
8. Interviewer characteristics	What characteristics were reported about the inter viewer/facilitator? e.g. Bias, assumptions, reasons and interests in the research topic	The interviewer had no prior experience of living libraries in any capacity and bias was mitigated through feedback from an advisory group with diverse experiences of living libraries (page 5 lines20-25)
Domain 2: study design		
Theoretical framework		
9. Methodological orientation and Theory	What methodological orientation was stated to underpin the study? e.g. grounded theory, discourse analysis, ethnography, phenomenology, content analysis	This study was underpinned by a realist methodological orientation (page 4, line 35)
Participant selection		

For peer review only

TITLE: Designing a Library of Lived Experience for Mental Health: integrated realist synthesis and experience-based co-design study

10. Sampling	How were participants selected? e.g. purposive, convenience, consecutive, snowball	Interviewees and workshop participants were purposively sampled based on their experience of living libraries (interviews, page 5, line 16) and mental health experiences (workshops, page 6, line 14-15)
11. Method of approach	How were participants approached? e.g. face-to-face, telephone, mail, email	Digital study advertisements (page 6, line 12)
12. Sample size	How many participants were in the study?	7 interviews (page 5, line 15) and 31 workshop participants (page 7, line 14)
13. Non-participation	How many people refused to participate or dropped out? Reasons?	Workshop retention is reported in Table 1 (page 8, line 22) – reasons for non-attendance were not recorded
Setting		
14. Setting of data collection	Where was the data collected? e.g. home, clinic, workplace	Data was collected online via video call. Page 5, line 19 (interviews), page 6, line 21 (workshops)
15. Presence of non-participants	Was anyone else present besides the participants and researchers?	No other people were present for interviews. Workshops included several co-facilitators described on page 6, lines 17-18 workshops).
16. Description of sample	What are the important characteristics of the sample? e.g. demographic data, date	Workshop demographics are provided in supplemental file 6. Interviewee demographic characteristics were not recorded.
Data collection		
17. Interview guide	Were questions, prompts, guides provided by the authors? Was it pilot tested?	Interview topic guide is provided in supplemental file 2.
18. Repeat interviews	Were repeat inter views carried out? If yes, how many?	No
19. Audio/visual recording	Did the research use audio or visual recording to collect the data?	Interviews were audio recorded (page 5, line 19). Workshops were not audio recorded in order to promote participants' feelings of safety in engaging with group discussions about mental health.
20. Field notes	Were field notes made during and/or after the interview or focus group?	Researcher field notes were generated during co-design workshops (page 6 lines 29). Not applicable to interviews

For peer review only

TITLE: Designing a Library of Lived Experience for Mental Health: integrated realist synthesis and experience-based co-design study

21. Duration	What was the duration of the interviews or focus group?	Specific online call duration was not recorded.
22. Data saturation	Was data saturation discussed?	No – data saturation is typically not discussed in this form of analysis
23. Transcripts returned	Were transcripts returned to participants for comment and/or correction?	No – however, analytic outputs were (page 6, lines 33-36)
Domain 3: analysis and findings		
Data analysis		
24. Number of data coders	How many data coders coded the data?	3 – PM, FL, and RJ (Page 6, lines 5-6)
25. Description of the coding tree	Did authors provide a description of the coding tree?	No
26. Derivation of themes	Were themes identified in advance or derived from the data?	From the data (page 5, lines 3-5)
27. Software	What software, if applicable, was used to manage the data?	NVivo 12 (page 6, line 31)
28. Participant checking	Did participants provide feedback on the findings?	Yes – written and verbal feedback from an advisory group (page 56, lines 33-36)
Reporting		
29. Quotations presented	Were participant quotations presented to illustrate the themes/findings? Was each quotation identified? e.g. participant number	No – however, deidentified workshop data were used to illustrate findings
30. Data and findings consistent	Was there consistency between the data presented and the findings?	In order to demonstrate this consistency, findings are presented with reference to extracts of underlying data from relevant literature and workshops (Table 2 – page 11, line 11)
31. Clarity of major themes	Were major themes clearly presented in the findings?	A detailed description of each major theme, or context-mechanism-outcome configuration (CMO), is presented (page 8 line 8 – page11 line 9)
32. Clarity of minor themes	Is there a description of diverse cases or discussion of minor themes?	Not applicable to all themes, however, diverse experiences of safety and potential harms are described in CMO 1 (page 8 line 10 – page 9 line 10)

For peer review only

TITLE: Designing a Library of Lived Experience for Mental Health: integrated realist synthesis and experience-based co-design study

Adapted from: Tong, A., Sainsbury, P., & Craig, J. (2007). Consolidated criteria for reporting qualitative research (COREQ): a 32-item checklist for interviews and focus groups. *International journal for quality in health care*, *19*(6), 349-357.

For peer review only

TITLE: Designing a Library of Lived Experience for Mental Health: integrated realist synthesis and experience-based co-design study

Supplemental file 2 – Interview topic guide

The topic guide should be amended to reflect the interviewees' experiences of living libraries as books/readers/organisers/researchers. In section 1, focus on developing an understanding of participant experiences by using open ended, exploratory questioning. In section 2, if applicable, use the developing programme theory to further explore participants' experiences with reference to the specific hypotheses being tested at that stage of theory development.

Section 1: Participant experiences of living libraries

Suggested topics and example questions (select as appropriate). These topics remain flexible as per the semi-structured interview method.

Topic 1: Type of event and context

- Did you attend as a book/reader/organiser?
- What was the purpose of the living library event you attended/organised?
- How did you find out/how were you recruited/how did you recruit for the event?
- What was the format of the event? In-person/online? Time restricted conversations? One off or recurring events?
- Were you given instructions prior to the event/did you provide training for attendees?

Topic 2: Sharing experiences

For peer review only

TITLE: Designing a Library of Lived Experience for Mental Health: integrated realist synthesis and experience-based co-design study

- Did you have any views/expectations about sharing/hearing a story before the event?
- How did you find out about the books that were available to speak with/provide options for readers?
- How did you decide who to speak to/when?
- What was the format of the discussions?

Topic 3: Impacts

- How did you experience sharing your story/hearing someone else's story?
- What was it like speaking with someone about [the topic] in particular?
- Were there unexpected effects of taking part?
- What effect (if any) did the living library event on your views of the topics discussed, and why?

Section 2: Theory testing

Following the guidance for realist interviews provided by Manzano (2016), prior to each interview, consider how the current programme theory can be further understood with reference to context, mechanism and outcome. Adapt the interview questions depending on the stage of theory development: in early interviews make greater use of general and exploratory questions to inform tentative theories; later, theory refinement focused interviews should increase in specificity and may draw on specific examples/cases to illustrate CMOs in discussions with participants; theory consolidation focuses on refining existing hypotheses, for example, how CMOs may vary depending on different stakeholder perspectives and how CMOs may link together across the programme theory.

For peer review only

TITLE: Designing a Library of Lived Experience for Mental Health: integrated realist synthesis and experience-based co-design study

Note: examples of how CMOs may be developed with a focus on theory development (adapted from Manzano, 2016):

Exploring context:

What characteristics of the living library location contribute to people feeling able to share their stories?

Looking for mechanisms:

How do you think the living library event influenced the attitudes of those who took part?

Looking for intended and unintended outcomes:

In your opinion, how should participants be supported when asked to share their experiences as part of a living library?

References

Manzano, A. (2016). The craft of interviewing in realist evaluation. *Evaluation*, *22*(3), 342-360.

For peer review only

TITLE: Designing a Library of Lived Experience for Mental Health: integrated realist synthesis and experience-based co-design study

Supplemental file 3 - Initial programme theories

1. Readers – hearing stories about mental health

Intended impacts

1.1 Reduced isolation - If readers hear stories that resonate with their own experiences of mental health difficulties, then they will feel that their experiences are shared, thus normalising their experience and reducing their sense of social isolation. This shared experience may make them feel they are more likely to be understood by the book, and by others with similar experiences, and therefore more able to talk about their mental health difficulties with others, promoting social engagement and help-seeking.

1.2 Tailored learning - If readers take part in a library that facilitates synchronous interaction, then they will be able to ask the questions about mental health that they want answered, increasing the relevance of the information shared by the book to their personal experience and individual learning needs. This will mean readers are better informed and have the information they need to manage their own mental health difficulties.

1.3 Challenging preconceptions - If readers interact with a living book who through their participation in the library challenges the reader's negative preconceptions or stigma regarding mental health, for example that it is not possible to live well with a mental health problem, then contact with the book will disconfirm these preconceptions, increasing hope for their own recovery.

1.4 Connecting to the individual - If the reader engages with a book in a synchronous, face-to-face interaction, then this intimate interpersonal context facilitated will promote empathy with the book as their storytelling will be perceived as more authentic and emotive, facilitating other intended impacts (such as learning, social connection, and hope for recovery).

1.5 Connecting to the story - If the reader interacts with a book whose story involves a compelling narrative about their mental health difficulties, then the reader will be more engaged by their story, making the story more likely to be internalised and remembered, facilitating other impacts such as lasting change in perspective. This impact will be greater in a context where shared life experiences create personal resonance with the story.

Unintended impacts

For peer review only

TITLE: Designing a Library of Lived Experience for Mental Health: integrated realist synthesis and experience-based co-design study

1.6 Exposure to distressing stories - If the reader interacts with a book who recalls distressing aspects of their experiences and/or who becomes distressed when doing so, then the reader may be at increased risk of themselves becoming distressed.

1.7 Interpersonal tension - If the reader engages with a book who they perceive holds views that they find objectionable, then there is an increased risk of interpersonal tension and possible subsequent distress is increased The reader is also unlikely to identify with the book, and more likely to actively reject any strategies or ideas that the book offers, so will not learn anything of value and feel they have wasted their time. This could make them less likely to engage with other forms of peer-based support in future.

1.8 Negative social comparison - If the reader engages in negative social comparison, for example perceives that they are unlikely to achieve the level of recovery experienced by the living books at the library, then they may feel dejected and less hopeful about their own recovery, and therefore less likely to engage in recovery focussed activities. This is particularly likely to happen if the books are selected for “success” (or “recovery”) stories only.

2. Living ‘books’ – telling stories about mental health

Intended impacts

2.1 Using experiences to help others - If living books tell their stories of mental health difficulties to readers who they feel have been derived benefit from hearing their experiences, those telling their stories will derive satisfaction from their participation contributing to a sense of personal meaning.

2.2 Developing personal understanding - If living books tell their stories of mental health difficulties, then the process of recalling and communicating their story in a safe environment may facilitate the development of new understandings of their previous experiences, contributing to greater awareness of their own mental health.

2.3 Social inclusion - Where living books are encouraged to use their mental health experiences as part of a mental health programme to help others, books may begin to value and drawn on these experiences to achieve personal goals such as greater social engagement, thus promoting social inclusion and involvement in other activities that invite them to draw on their lived expertise.

2.4 Being empowered - If living books are supported to tell their stories of mental health difficulties in a way that is authentic to their experience and aligns with their motivation for sharing their story, then they will experience a sense of empowerment because the process of storytelling will promote autonomy over expression of an experience that in society more broadly is often stigmatised and supressed.

For peer review only

TITLE: Designing a Library of Lived Experience for Mental Health: integrated realist synthesis and experience-based co-design study

Unintended impacts

2.5 Recalling distressing experiences - If living books tell their experience of mental health difficulties, without adequate training and support, then the process of recalling painful experiences may trigger distress because they are still emotionally processing these experiences.

2.6 Devaluing of lived experiences - If living books attempt to tell their experiences to a reader who is perceived to be disinterested, disrespectful or insensitive, then they will be at increased risk of feeling that their lived experiences of distress have devalued contributing to disengagement from the library.

3. Organisation and support

3.1 Balancing conversational openness and risk of harm - Impacts of a living library for mental health will be moderated by organisational aims, including the purpose of the library, and organisational culture, including the extent to which lived experience of distress is deemed to be an important resource for shared learning. These factors will determine how organisers, or 'librarians', understand the need to balance the facilitation of open conversations about mental health with the need to restrict or structure conversations to reduce perceived risk to participants. This will determine when and how they intervene to support conversations within the library.

3.2 Curation and commodification - If the stories selected for the library, and the way they are told (curation), is perceived by books to be prescriptive to the extent that their stories are no longer authentic expressions of their lived experience, then books may perceive that their stories of mental health experiences are being commodified – used for a purpose that is incongruent with their motivation for participating in the library - and thus devalued. This is likely to lead to them withdrawing participation in the library.

3.3 Location facilitates engagement - If the physical location in which the library is held facilitates a feeling of psychological safety, for example within a space that means personal disclosure remains private and where participants are not overlooked by the public, then both books and readers will be more likely to engage in open and engaging conversations because they will feel better able to immerse themselves in conversation without distraction or concern over confidentiality.

3.4 Perceived emotional support - Where books and readers perceive that the library provides or will provide sufficient emotional support to address any potential distress, they will feel safe and held, and so and will be more open to engaging with potentially distressing conversations about mental health.

For peer review only

TITLE: Designing a Library of Lived Experience for Mental Health: integrated realist synthesis and experience-based co-design study

3.5 Setting expectations - Impacts of a living library are likely to depend on the expectations of both books and readers. Where it is made clear to participants what is expected of them within the library and any boundaries in place, participants will be better equipped to manage any difficulties that may arise and/or engage with the programme as intended, because they will understand the purpose and function of the library.

3.6 Setting ground rules – Where the 'librarians' or organisers set clear guidelines for engaging in conversations about mental health, including the necessity of communicating respectfully and what to do if difficult conversation do arise, both books and readers will feel empowered to engage in more open and sincere conversation.

For peer review only

TITLE: Designing a Library of Lived Experience for Mental Health: integrated realist synthesis and experience-based co-design study

Supplemental file 4 – search strategies

Search 1: Initial search for living library-related literature.

Searches for literature related to living libraries was intentionally broad and targeted academic and grey literature databases in health, social care, and education. An initial search retrieved articles from database inception to February 2022. A second, updated search using the same search strategy retrieved evidence from February 2022-March 2023.

Databases:

Published literature:

- Web of Science
- Scopus
- PsycINFO
- MEDLINE
- CINAHL
- Embase
- SocINDEX
- Social Care Online
- British Education Index
- Education Abstracts
- Library, Information Science & Technology Abstracts

Grey literature:

- ProQuest
- British Library
- International Clinical Trials Registry Platform
- Overton.io
- Bielefeld Academic Search Engine
- Google.com (top 100 search results)
- Scholar.google.com (top 100 search results)

Search terms:

“living librar*” OR “human librar*” applied to ‘title’, ‘abstract’, and ‘keywords’ fields (if available)

Eligibility criteria

- Published (including academic articles and book chapters) or grey literature (including theses, blogs, reports, conference documents and policy documents)
- Available in English
- 2000 (inception of the programme) to date
- Specifically relates to a human or living library event
- Text only

For peer review only

TITLE: Designing a Library of Lived Experience for Mental Health: integrated realist synthesis and experience-based co-design study

Search 2: Targeted search for theories related to psychological safety.

This search was conducted in June 2022 on Web of Science. Resources available for the review precluded full review of the research literature and as such, a large multi-disciplinary database was selected for this search. Its aim was to identify English language articles in health-related research that included middle range theories related to the concept of psychological safety. Search terms were informed by the BeHEMoTh framework [24] and combined with the 'AND' Boolean operator:

Behaviour of interest: "psychological safety"

Health context: "health"

Exclusions: n/a

Models or **th**eories: "model* or theor* or concept* or framework*"

The search returned 290 articles. Title and abstracts were screened by PM. Articles that appeared relevant to the research question and suggested that they may include a middle range theory were flagged for full-text screening. Twenty-nine articles were read by PM and RJ who held collaborative discussions, through which one article was identified as including a middle range theory of psychological safety relevant to the developing programme theory.

For peer review only

TITLE: Designing a Library of Lived Experience for Mental Health: integrated realist synthesis and experience-based co-design study

Supplemental file 5 - Characteristics of included studies

Author(s), year	Document type	Setting, Country	Aim/objective	Book focus (General/focused)	Readers sample
Ashmore (2010)	Journal article	n/a, Canada	'…provides a discussion of the development and implementation of living library programs in the context of creating and fostering community engagement in the library.'	n/a	n/a
Bagci & Blazhenkova (2020)	Journal article	University, Turkey	'…tested the effectiveness of HL as a direct intergroup contact intervention on affective (trust, empathy), cognitive (knowledge), and behavioural (e.g., willingness to talk to outgroup members) aspects of outgroup attitudes'	General. Books included 'Schizophrenic'	University students
Blizzard, Becker, &Goebel, 2018	Journal article	University, Canada	'…describe the Augustana human library and how the team has used the event to take a new approach to teaching information literacy.'	Focused. Books related to Women's Studies	University students
Chung, Tse, & Tasha, 2022	Journal article	Educational setting, Hong Kong	'This study aimed to test the effectiveness of a human library intervention at enhancing MHL [mental health literacy]'	Focused. Books on mental health (bipolar disorder and schizophrenia)	Young adults in tertiary education
Constable & Harris, 2008	Journal article	Public libraries, UK	'… to explore a community development model of Living Library within public libraries in England.'	General	Public
Copenhaver & Koclanes, 2019	Journal article	University	'Raise awareness of social justice issues and to expose students to issues present in our local community'	General	University staff/students and the public
Dreher & Mowbray	Research monograph	Public and third sector organisations, Australia	'…comprehensive and independent analysis of Human Libraries (formerly Living Libraries) in Australia'	Varied. Typically General	Varied. Typically public.
Fortune & Leighton, 2022	Journal article	Community support centre for people with mental illness	'we report on in this paper focused on the experiences of individuals living with mental illness who became living books for an initiative aimed at addressing stigma by dispelling stereotypes related to mental illness through inclusionary dialogue.'	Focused. Mental health	Under-graduate therapeutic recreation students
Gamtso, Mannon, & Whipple, 2017	Book chapter	High school, university and public libraries, USA	'describe the planning and implementation of the New Hampshire program…and a discussion of the New Hampshire Human Library's impact on participants' learning.	Varied. Typically General	Students and/or the public

TITLE: Designing a Library of Lived Experience for Mental Health: integrated realist synthesis and experience-based co-design study

Garbutt, 2008	Conference paper	n/a, Australia	'The purpose of this paper is to begin this theoretical discussion of living libraries and their aims.	n/a	n/a
Giesler, 2021	Journal Article	University, USA	…explored two research questions: How do those in the role of enlisted participants (Books) describe the HL [human library] experience? and What potential does the HL have to create IGD [intergroup dialogue] in the social work classroom?'	General	Social work students
Giesler 2020	Magazine article	University, USA	Discusses 'What makes the Human Library so powerful? How can social work professionals and educators use it to weave together the profession's core values?'	General	University students
Giesler & Juarez, 2019	Journal Article	University, USA	'(1) to assess the power of the Living Library experience to bring people from disparate backgrounds …(2) to discern how the Living Library project builds community…(3) to add to the literature outlining potential benefits and risks of veterans who tell their stories of military service.	Focused. Books on veterans' experiences	Varied
Groyecka et al., 2019	Journal Article	Public library, Poland	…examined the effectiveness of the Human Library (held Wroclaw, Poland) in reducing social distance towards Roma, Muslims, dark-skinned, and transgender people, as well as in decreasing homonegativity.	General. Books included titles related to bulimia and anorexia, and schizophrenia.	Public
Hewlin-Via et al., 2021	Research poster	University, Australia	'To evaluate the changes in students' cultural awareness and sensitivity after attending the HL. To understand the perspective of student OTs [Occupational Therapists] in participating in the HL [Human Library] …'	General. Books included growing up with mental illness	Student occupation al therapists
Huang & Dobreski, 2017	Conference paper	Public and university libraries, USA	'We present how libraries have attempted to utilize technologies and leverage community support to enable human library events.'	General	Students and/or the public
Inkster et al., 2016	Research report	Hospital, UK	'to challenge prejudice in healthcare'.	General. Books included 'mental health problems'	Healthcare staff
Kudo et al., 2011	Conference paper	University, Japan	'…provides a first-person narrative of preparation, challenges and success of a one-day Human Library administered by 30 undergraduate students and their supervisor at a Japanese university.'	General	Public
Kwan, 2020	Journal article	Mental health service centre, Hong Kong	'explores the relevance and usefulness of this approach [living library] in a mental health setting'	Focused. Books on mental health	People in recovery and other community members

For peer review only

TITLE: Designing a Library of Lived Experience for Mental Health: integrated realist synthesis and experience-based co-design study

Lam, Wong & Zhang, 2023	Journal article	n/a	'…his article aims to review the current body of knowledge on HL by conducting a systematic narrative review on the existing literature.'	Varied	Varied.
Little et al., 2011	Implementation guide	Various, Council of Europe	'This guide is intended to provide support and direction to Living Library organisers in Europe.'	General	Varied. Typically public.
Michalak et al., 2019	Journal article	Online, Canada	'… (1) to create an innovative Web-based Bipolar Wellness Centre, (2) to conduct a mixed-methods (i.e., quantitative and qualitative) evaluation to assess the impact of different sorts of engagement (i.e., knowledge translation [KT]), and (3) to support engagement with the self-management information in the Bipolar Wellness Centre'	Focused. Books were people with experience of bipolar disorder.	People with bipolar disorder
Orosz et al., 2016	Journal article	High School, Hungary	'assesses the effectiveness of the Living Library intervention and tests whether and how perceived peer prejudices moderate its effectiveness in reducing prejudice toward Roma and LGBT people'	Focused. Books were Roma and members of the LGBT community.	High school students
Pardasani & Rivera, 2017	Thesis	University, Finland	'to serve as a guidebook in itself as a reference for the implementation guidelines, training and promotional material and a resource material for the future Human Library events at DIAK or any other social work institutions.'	General. Books included people with anxiety and depression.	Students
Pratchett, 2019	Blog	Hospital, UK	'to bring staff from different areas of the trust together and enable them to hear the lived experience of colleagues in their own words.'	General.	Healthcare staff
Russell & Linsley, 2011	Journal article	University, UK	'The project centred on the running of a 'human library' event in which adult nursing and social work students engaged with mental health service users through an open forum'.	Focused. Mental health service users	Student nurses and social workers
Sen et al., 2016	Journal article	University, UK	'describes the running of four Living Libraries on a UK postgraduate social work course'	General & focused, with Books related to the experience of social care.	Student social workers
Sen et al., 2021	Book chapter	Universities, UK & Finland	'Two approaches are presented from two universities … In the University of Sheffield (UK), people with experiences of social work services, teachers and students have developed living libraries as a regular feature of the syllabus for a qualifying master's degree programme in social work since 2014. In the Diaconia University of Applied Sciences (Diak, Finland), bachelor-level students in social services initiated and organised a Human Library event…'	General & focused, with stories related young people's experiences of social care services	Student social workers/Public

For peer review only

TITLE: Designing a Library of Lived Experience for Mental Health: integrated realist synthesis and experience-based co-design study

Watson 2015	Thesis	University, Australia	'The research question is: What does an examination of Human Library inform us about how people can challenge prejudice and increase respect for difference as a means for promoting humans' rights and freedoms?'	General	Public

For peer review only

TITLE: Designing a Library of Lived Experience for Mental Health: integrated realist synthesis and experience-based co-design study

Supplemental file 6 – Participant demographics

ID	Age group	Gender identity	Ethnic group	Current or previous mental health experience
1	51-60	Female	White/White British	Carer; voluntary sector staff
2	31-40	Male	Black/African/Caribbean/ Black British	Mental health service user
3	51-60	Female	White/White British	Mental health service user; carer; voluntary sector service user; voluntary sector staff
4	41-50	Male	White/White British	Mental health service user; mental health service staff; carer; voluntary sector staff
5	61-70	Male	White/White British	Mental health service user; voluntary sector staff
6	18-30	Female	Asian/Asian British	Mental health service user; carer; voluntary sector service user
7	51-60	Male	White/White British	Mental health service user
8	41-50	Male	White/White British	Mental health service staff
9	31-40	Female	White/White British	Mental health service user; mental health service staff
10	18-30	Female	White/White British	Carer; voluntary sector staff
11	51-60	Male	White/White British	Mental health service user; mental health service staff; carer
12	71-80	Male	White/White British	Mental health service user
13	41-50	Male	White/White British	Mental health service user; mental health service staff
14	31-40	Female	Other ethnic group	Voluntary sector service user
15	41-50	Male	White/White British	Mental health service user
16	51-60	Male	White/White British	Carer; voluntary sector staff
17	31-40	Female	White/White British	Mental health service user; carer; mental health service staff
18	18-30	Male	Black/African/Caribbean/ Black British	Mental health service user
19	31-40	Female	White/White British	Mental health service user; mental health service staff; carer
20	31-40	Male	White/White British	Mental health service user; voluntary sector staff
21	41-50	Female	Asian/Asian British	Carer
22	61-70	Male	White/White British	Mental health service user; voluntary sector staff
23	41-50	Female	White/White British	Carer
24	41-50	Female	Mixed/multiple ethnic groups	Mental health service user
25	61-70	Female	White/White British	Mental health service user; voluntary sector service user
26	41-50	Male	White/White British	Mental health service user
27	51-60	Female	White/White British	Mental health service user; carer; voluntary sector service user
28	18-30	Female	White/White British	Mental health service user
29	31-40	Male	Other ethnic group	Mental health service user; voluntary sector service user
30	51-60	Female	Other ethnic group	Mental health service user

For peer review only

TITLE: Designing a Library of Lived Experience for Mental Health: integrated realist synthesis and experience-based co-design study

31	51-60	Female	White/White British	Mental health service staff; carer

For peer review only
